# GenPath-PPH: Integrating gene expression and pathway networks via persistent path homology enhances detection of disease-relevant pathways

**DOI:** 10.1016/j.csbj.2025.11.018

**Published:** 2025-11-26

**Authors:** Muhammad Sirajo Abdullahi, Rosario Michael Piro, Apichat Suratanee, Kitiporn Plaimas

**Affiliations:** S2001037025004891-ed7c4ff3fefcc30337ff24b82fb00194 Advanced Virtual and Intelligence Computing (AVIC) Center, Department of Mathematics and Computer Science, Faculty of Science, Chulalongkorn University, 10330, Bangkok, Thailand; S2001037025004891-3437f72553bf6ee5a6a3e68d3c0a1706 Department of Mathematics, Faculty of Physical and Computing Sciences, Usmanu Danfodiyo University, Sokoto, 840104, Sokoto, Nigeria; S2001037025004891-67a872f1ff1e76a55273f68d9c11182a Dipartimento di Elettronica, Informazione e Bioingegneria, Politecnico di Milano, 20133, Milan, Italy; S2001037025004891-b8b4ab82e54ec60cd8739c46dbc3c0a3 Department of Mathematics, Faculty of Applied Science, King Mongkut’s University of Technology North Bangkok, 10800, Bangkok, Thailand; S2001037025004891-8ddc786bc45e3345ae84aa7865db7cf7 Intelligent and Nonlinear Dynamic Innovations Research Center, Science and Technology Research Institute, King Mongkut’s University of Technology North Bangkok, 10800, Bangkok, Thailand; S2001037025004891-740ce5ae7d9fcc2b20002d1972945f62 Centre of Excellence in Mathematics, Ministry of Higher Education, Science, Research, and Innovation, National University of Sciences, 10400, Bangkok, Thailand

**Keywords:** Persistent path homology, Betti numbers, Topological data analysis, Gene expression, Pathway networks, Hepatocellular carcinoma

## Abstract

Identifying disease-relevant biological pathways is critical for understanding the molecular mechanisms underlying specific disease phenotypes. Traditional techniques, such as gene set analysis, often neglect topological interactions among genes within actual biological pathways. To address this limitation, we introduce GenPath-PPH, a novel framework that integrates gene expression data with directed biological pathway networks using persistent path homology (PPH), a topological tool for analyzing directional relationships. GenPath-PPH tracks changes in correlation strength between interacting genes across two conditions (e.g., disease and control) and interprets these differences as topological, disease-related alterations in the pathway network. Within pathways, it identifies changes in connected components (co-expression clusters) as well as in higher-order structures (directed cycles), which are not detectable by conventional homology methods. By combining connectivity and cyclic features, GenPath-PPH highlights significantly altered pathways, using permutation testing to assess statistical significance. When applied to peripheral blood mononuclear cell (PBMC) samples from hepatocellular carcinoma (HCC) patients, GenPath-PPH not only identifies well-known cancer-associated pathways (e.g., JAK-STAT signaling, p53 signaling and the pentose phosphate pathway) in accordance with other techniques, but also reveals additional pathways (e.g., NF-κB signaling, sphingolipid signaling and aminoacyl-tRNA biosynthesis) that are either missed by other techniques, despite their known relevance to HCC, or represent novel candidate pathways for experimental evaluation. Our work bridges network topology and biological function, offering a new analytical approach capable of uncovering previously overlooked connections between topological structure and functional activity.

## Graphical Abstract

**Figure:**
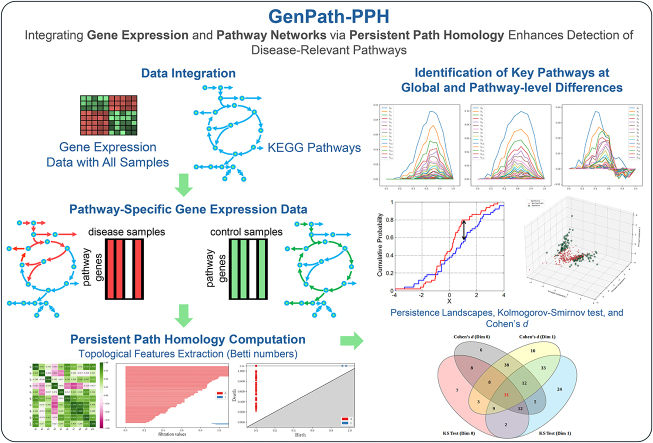

## Nomenclature

**Acronyms**
BH
: Benjamini Hochberg
CDF
: Cumulative distribution function
FDR
: False discovery rate
GSEA
: Gene set enrichment analysis
HCC
: Hepatocellular carcinoma
HGEA
: Hypergeometric enrichment analysis
KS
: Kolmogorov-Smirnov
KGML
: KEGG markup language
KEGG
: Kyoto encyclopedia of genes and genomes
PBMC
: Peripheral blood mononuclear cells
PD
: Persistent diagram
PL
: Persistent landscape
PH
: Persistent homology
PH-TD
: Persistent homology with topological descriptors
PPH
: Persistent path homology
PCA
: Principal component analysis
TDA
: Topological data analysis
TPM
: Transcripts per million

## 1. Introduction

Identifying deregulated biological pathways is vital for revealing processes behind disease progression, facilitating biomarker discovery, and identifying therapeutic targets [1,2]. Pathway analysis aims to identify biologically relevant processes from gene expression data, by statistically evaluating expression differences between disease and control states. However, traditional methods, such as statistical, enrichment-based approaches, rely on expression changes of sets of individual genes (e.g., differential expression) while ignoring network topology and dynamic gene relationships [3–6]. This shortcoming limits their ability to capture the cooperative interactions driving biological processes [7].

Static topological methods, such as those using centrality measures or positional importance, partially address this gap by mapping measured gene expression levels to fixed network structures [8–12]. However, they cannot model dynamic, multi-scale topological changes (i.e., changes observed by tracking how network topology arises from isolated genes to a full pathway network where edges are added based on decreasing gene correlation strength). This is a critical limitation in contexts where subtle network rewiring may signal key biological transitions [13].

Topology, as a branch of mathematics that examines the connectivity and shape of structures, focuses on relationships between components rather than exact distances or lengths. In biology, this is particularly useful for analyzing gene interaction networks, where the directional influence of one gene on another (e.g., activation or repression) may define the system’s functional architecture more critically than individual expression levels alone. It is therefore not surprising that in recent years, topological data analysis (TDA) has gained attention for its ability to capture the structural complexity in biological networks [14,15].

In contrast to traditional methods, TDA is uniquely suited for studying dynamic pathway topology across biological conditions (e.g., disease vs. healthy control) because it models pathway interactions at multiple scales. Still, integrating gene expression with pathway networks remains challenging because conventional tools often fail to detect subtle yet biologically meaningful topological changes [16].

Homology, a key concept in topology, identifies and counts topological features such as clusters (connected components; $\beta_{0}$) and loops (cycles; $\beta_{1}$) by counting “holes" in an object in a topological space, such as a point cloud representing genes and their expression levels in a set of tissue samples.

Advances in computational topology, including persistent homology (PH), have introduced tools for analyzing multi-scale topological features [17,18], such as connected components and loops, across different filtration values (i.e., threshold parameters for an appropriate distance measure, that control at which level network connectivity is observed).

PH, like classical homology, ignores the directionality of the edges in a network, a key property of biological pathways (e.g., directed regulatory cascades). Path homology addresses this by modeling directed edges, and persistent path homology (PPH) further extends this to track directional topological features across filtration values [19,20]. PPH has been applied to analyze topological features in other datasets, successfully capturing structural information in directed graphs [21,22].

For example, in the p53 signaling pathway, the sequence of interactions (TP53 ↦ BAX and TP53 ↦ SIVA1 ↦ BCL2 ↦ BAX) forms a path-like cycle, where multiple directed interactions converge on BAX [23,24]. This structure represents a regulatory feed-forward pattern—highlighting alternative regulatory routes in apoptosis—that conventional PH may overlook or misinterpret.

In this study, we present GenPath-PPH (Gene expression and Pathway network integration using Persistent Path Homology), an extension of PPH suitable for biological network analysis. GenPath-PPH integrates the Kyoto Encyclopedia of Genes and Genomes (KEGG, [25]) pathway information with pathway-specific gene expression profiles using PPH. This offers a dynamic and multi-scale perspective of how pathway structures adapt across varying biological conditions. The integration overcomes the limitations of static methods by enabling the analysis of pathway activity in response to changing conditions.

The remainder of this paper is organized as follows: Section 2 presents some background on PPH and its biological interpretation, while the formal mathematical definitions are provided in Appendix A. Section 3 describes the datasets and methods used in the study, and Section 4 explains the GenPath-PPH workflow, including a toy example that illustrates the procedure and the major differences from classical PH. Section 5 reports the application of GenPath-PPH to peripheral blood samples of hepatocellular carcinoma (HCC) patients. Finally, Section 6 summarizes the main findings and outlines future research directions.

## 2. Background

**Fig. A.10.**
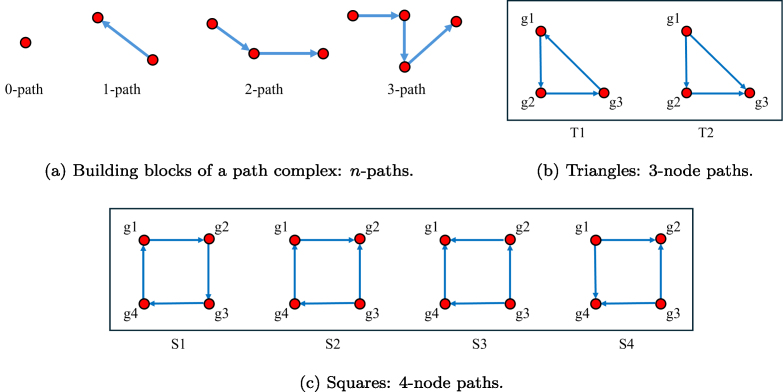
Illustration of basic path complex structures. **(a)** Examples of n-paths (n=0,1,2,…). **(b)** Triangular configurations: T1 forms a 1-cycle ($\beta_{1}=1$) while T2 does not ($\beta_{1}=0$). **(c)** Square configurations: S1, S2, and S4 contain one hole ($\beta_{1}=1$), whereas S3 has none ($\beta_{1}=0$). These highlight how path homology distinguishes between directed patterns that classical homology treats equivalently.

The concept of path homology provides a mathematical framework capable of incorporating directional information, making it particularly well-suited for analyzing biological systems such as gene regulatory networks, signaling cascades, and metabolic pathways. It is built on a mathematical structure called a *path complex* [26], which records all valid sequences of connected vertices (paths) within a network—ranging from individual nodes (0-paths) and edges (1-paths) to higher-dimensional paths (e.g., 2- or 3-paths composed of consecutive directed edges; see Fig. A.10(a)). This allows the representation of complex biological relationships where both direction and order of interactions are meaningful. While particularly useful for studying directed pathways, path homology can also be applied to undirected networks or more abstract systems. A concise description of the formal construction of path complexes and their associated homology groups is provided in Appendix A.

PPH extends path homology to study how different topological features of a directed network persist as connectivity thresholds (filtration values) between nodes are gradually increased—from a minimum, where no edges exist (only individual nodes), to a maximum, where all network edges are included (full connectivity). The persistence of a topological feature corresponds to the range of filtration values over which it exists, from its first appearance to when it merges with another feature. How the connectivity between two linked nodes is measured for this purpose can be adapted to the overall goal of the analysis and the underlying data type. In GenPath-PPH, we define filtration values based on gene co-expression measurements—where a stronger co-expression is observable at lower filtration levels and a weaker co-expression requires higher levels for edges to be included (see Section 3.4 for more details)—and identify stable topological features such as clusters (connected components, corresponding to $\beta_{0}$) and cycles (loops, corresponding to $\beta_{1}$) that persist across multiple scales. This makes the approach particularly well suited for directed biological networks such as KEGG pathways.

Betti numbers ($\beta_{n}$) are used to quantify topological features of different dimensions n within these networks. For instance, $\beta_{0}$ counts connected components (e.g., clusters of co-expressed or functionally related genes), $\beta_{1}$ counts independent cycles (e.g., recurrent or feedback-like gene interactions), and higher $\beta_{n}$ (n≥2) describe more complex void-like structures. Since most biological pathways don’t have higher-order structures, we focus on $\beta_{0}$ and $\beta_{1}$. For example, in the p53 signaling pathway, a non-zero $\beta_{1}$ indicates persistent cyclic gene interactions such as TP53 ↦ BAX, TP53 ↦ SIVA1 ↦ BCL2 ↦ BAX, while a decreasing $\beta_{0}$ reflects the merging of smaller co-expression clusters as the filtration value increases.

Plotting Betti numbers as a function of increasing filtration values generates Betti curves that illustrate how topological features appear, disappear or persist as network connectivity strengthens. Peaks in $\beta_{0}$—representing the number of connected components—typically occur at low filtration values when interacting linked gene pairs remain separate due to weaker co-expression. As filtration values increase, edges between genes with weaker co-expression are included, causing $\beta_{0}$ to decline as smaller components merge into larger ones. For $\beta_{1}$, peaks may occur at any point along the filtration scale. Early peaks indicate that the genes forming the cycles are strongly correlated, whereas later peaks suggest weaker correlations that delay the formation of such cycles until edges between those weakly correlated genes are incorporated into the network. Tracking Betti numbers across filtration values helps quantify persistent features that are more likely to be biologically relevant and important for understanding the pathway network. Topological features that have a long persistence tend to be associated with network patterns composed of highly correlated genes (appearing at low filtration values) and low correlation with other network components (merging only at high filtration values, e.g., to form higher-order structures).

## 3. Materials and methods

### 3.1. Gene expression dataset

The gene expression dataset we analyzed was from [27], which employed RNA-seq to profile gene expression patterns in peripheral blood mononuclear cells (PBMCs) from 17 HCC patients and 17 age-matched healthy controls. We normalized raw per-gene read counts using Transcripts Per Million (TPM) [28] to account for transcript length and sequencing depth, followed by log_2_ transformation to stabilize variance. Normalization and transformation were performed using the *edgeR* [29] R package (v 3.42.4, R version 4.3.0). For more detailed information on the RNA-seq data processing, we refer readers to our previous work [30].

### 3.2. Biological pathways and network data

We retrieved 251 metabolic and signaling pathways and their corresponding gene sets from the KEGG database [25] using the R packages *AnnotationDbi* v1.66.0 [31] and *org.Hs.eg.db* v3.19.1 [32] with R (v4.4.1). Pathway interactions (e.g., activations, inhibitions) were parsed from KEGG Markup Language (KGML) files downloaded from the KEGG website (https://www.genome.jp/kegg/pathway.html). The Bio.KEGG.REST module in Biopython v1.1.76 [33] for Python (v3.12.6) was used to convert KEGG gene IDs to NCBI gene symbols, ensuring consistency with the HCC gene expression dataset.

For each processed pathway, the obtained network contained one node for every gene involved in the pathway, and directed edges between genes were placed for activations, inhibitions, or other interactions defined in KEGG. In cases where a KEGG pathway defined nodes containing multiple genes (e.g., a group of genes performing a shared function), these were separated into individual nodes, while maintaining for each of the separated nodes all edges associated with the original group of genes. This step ensured that the final network accurately captured all individual gene interactions and maintained a one-to-one correspondence with the gene list used for gene expression analysis. Finally, each pathway network was described in the form of an adjacency matrix.

### 3.3. Representation of gene expression data

For each pathway and each health condition (HCC or control), the gene expression profiles (expression levels measured in the 17 corresponding PBMC samples) of all genes involved in the pathway were mapped to a 17-dimensional space to obtain a condition-specific point cloud. In this point cloud, each point corresponds to a gene node in the pathway network, and each dimension in the space reflects the gene expression levels in one of the 17 samples for that health condition.

Each obtained point cloud can be used for a PH analysis, or—jointly with the corresponding pathway network for which it was constructed—for a PPH analysis with GenPath-PPH. In both analyses, the topological properties of the two point clouds of a pathway, one for HCC and one for healthy controls, can be compared.

### 3.4. Distance metric and filtration

To prioritize biologically meaningful gene relationships, we replaced the Euclidean distance originally used by *PathHom* [22] with a correlation-based distance metric d=1−|ρ|, where ρ is the Pearson correlation coefficient. This coefficient is frequently used by the scientific community to determine the strength of co-expression between two genes [34]. This correlation-based distance metric ranges from 0 (perfect correlation, regardless of direction) to 1 (no correlation), helping to emphasize strong gene interactions while filtering noise from weak correlations.

Using the absolute value |ρ| aligns with the biological intuition that coordinated activity—whether positive or negative—can jointly drive pathway behavior. A negative correlation can be as biologically relevant as positive correlation, e.g., when one gene inhibits the activity of another. Thus, this formulation captures both co-activation and inverse regulatory patterns within pathway networks.

Alternative similarity measures, such as Spearman or partial correlation, or mutual information, can also be applied depending on the data characteristics and the type of dependencies of interest. In this study, we selected the Pearson-based metric for its interpretability and its established use in co-expression network analysis.

This distance metric was then used as the correlation threshold for filtrations (see Section 2). At very low filtration values f (close to zero), only the edges between the closest genes (d≤f, i.e., highly correlated) are taken into account to determine topological features in terms of Betti numbers. As f increases, edges between more distant genes (those representing weaker correlations) are progressively included, until at f=1 the entire network is considered. In this study, we increase the filtration values/thresholds stepwise from 0 to 1 with a step size of 0.01.

### 3.5. Global differences between health conditions

For each pathway, two series of Betti numbers (one for the control group and one for the disease group)—as a function of the filtration value, i.e., distance threshold ($f\in \{0.01\;k|k\in N_{0},k\leq 100\}$)—were obtained from PPH computations for both dimension 0 (connected components) and dimension 1 (cycles).

These series of Betti numbers were then converted to persistence diagrams (PDs; see Fig. 8 in Section 5 for examples), which are graphical representations of the topological features (such as connected components or cycles) and their persistence across the filtration scale. Each point in a PD represents a topological feature, with the x-coordinate indicating its birth time (when the feature first appears) and the y-coordinate indicating its death time (when the feature disappears or ceases to exist) during the filtration process.

The PDs were then transformed into persistence landscapes (PLs), which hierarchically rank topological features based on their prominence across filtrations. Each landscape function $\lambda_{k}$ represents the k-th most persistent feature, where $\lambda_{1}$ captures the most dominant structure (e.g., stable gene cluster or cycle), $\lambda_{2}$ the second most dominant, and so on.

PLs provide a computationally convenient way to summarize the persistence of topological features. PLs are sequences of piecewise-linear functions, making them faster to compute compared to PDs, and allowing arithmetic comparisons (such as addition, subtraction, and averaging) that are not possible with PDs. By using functional norms (sup-norm, 1-norm, and 2-norm) we can measure the maximum deviation, total absolute difference, and Euclidean distance of a PL, respectively. This makes it possible to perform meaningful comparisons between the PLs of different conditions (for example, disease versus control).

To assess global differences between conditions, PLs from all pathways were averaged for each specific condition group g and dimension n (connected components, $\beta_{0}$, or directed cycles, $\beta_{1}$). The average PL for dimension n in condition g is defined as:

$$\overset{―}{PL_{n}^{\text{g}}}=\frac{1}{k}\sum_{i=1}^{k}PL_{n}^{\left(i,\;\text{g}\right)}$$

where k is the total number of pathways, and $PL_{n}^{\left(i,\;\text{g}\right)}$ is the PL of the i-th pathway for the given condition group g.

The difference between the disease condition D and the control condition C was computed as:

$$\Delta PL_{0}=\overset{―}{PL_{0}^{\text{D}}}-\overset{―}{PL_{0}^{\text{C}}}\;\text{and}\;\Delta PL_{1}=\overset{―}{PL_{1}^{\text{D}}}-\overset{―}{PL_{1}^{\text{C}}}.$$

To evaluate whether the observed differences in PLs (ΔPL) were statistically significant, a permutation test was conducted separately for each homological dimension n (connected components and cycles). PLs from all pathways across both conditions were pooled, randomly reassigned into two groups matching the original number of PLs for each group, and used to recalculate the average PLs ($\Delta PL_{0}$ for connected components and $\Delta PL_{1}$ for cycles). This process was repeated 1000 times to generate a null distribution of randomized differences. For each iteration, the sup-norm (maximum deviation), the 1-norm (total absolute difference), and the 2-norm (Euclidean distance) were computed for $\Delta PL_{0}$ and $\Delta PL_{1}$. The differences observed from the true data were compared to this null distribution, and p-values were calculated as the proportion of permutations with differences greater than or equal to the observed values from the true data. Finally, p-values from all three norms were adjusted using the Benjamini–Hochberg method (BH [35]) to control the false discovery rate (FDR <0.05).

### 3.6. Pathway-level differences

The two series of Betti numbers obtained for each pathway, health condition and dimension of topological features (see Section 3.5) can also be used to explore pathway-level differences and identify specific pathways where topological differences distinguish between conditions, i.e., where the disease condition significantly affects the topological structure, for example, by increasing fragmentation (e.g., more isolated components indicating less correlation between genes) or enhancing cyclic interactions (e.g., stronger correlation between genes involved in directed cycles).

By applying the Kolmogorov-Smirnov (KS) test and Cohen’s d to the Betti series, we can identify the affected pathways and better understand how the condition influences the topology at a local level. To quantify these pathway-level differences, we therefore applied these two complementary metrics:

• *Kolmogorov-Smirnov (KS) test:* To compare the distributions of Betti numbers between control (C) and disease (D) groups across all filtration values, we applied the KS test (a non-parametric statistical method) using the Python package *SciPy* v1.14.1 [36]. The KS test quantifies the maximum divergence between the empirical cumulative distribution functions (CDFs) of two samples, making no assumptions about their underlying distributions.
  For a given pathway and dimension n ($\beta_{0}$: connected components, $\beta_{1}$: cycles), the KS statistic K is defined as:
  $$K=\underset{f}{\sup}|F_{C}(f)-F_{D}(f)|,$$
  where $F_{C}(f)$ and $F_{D}(f)$ are the empirical CDFs of Betti numbers for control and disease groups over the filtration values f, respectively, and $\underset{f}{\sup}$ denotes the supremum (greatest difference) across all filtration steps. A larger K value indicates greater separation between the distributions, suggesting potentially significant topological differences between conditions. For example, K>0.5 implies the disease group’s Betti numbers are stochastically dominant over the control group’s across filtrations. This helps identify pathways where topological features in the disease group are significantly different from those in the control group.
• *Effect size (Cohen’s* d*):* Cohen’s d quantifies the standardized difference in mean Betti numbers between the disease and control groups, providing insight into the magnitude of the topological differences. To calculate Cohen’s d, we first compute the mean Betti difference $\left(\mu_{\Delta }\right)$, which represents the average difference in Betti numbers across all filtration steps:
  $$\mu_{\Delta }=\frac{1}{\tau }\sum_{t=1}^{\tau }\beta_{n}^{C}(t)-\frac{1}{\tau }\sum_{t=1}^{\tau }\beta_{n}^{D}(t),$$
  where τ is the total number of filtration steps, and $\beta_{n}^{C}(t)$ and $\beta_{n}^{D}(t)$ denote the Betti numbers for the control and disease groups, respectively, at the t-th filtration step.
  Cohen’s d is then calculated as:
  $$d=\frac{\mu_{\Delta }}{s},$$
  where $\mu_{\Delta }$ is the mean Betti difference (average difference in Betti numbers across all filtration steps), and s is the pooled standard deviation of Betti numbers, computed as:
  $$s=\sqrt{\frac{(n_{1}-1)s_{C}^{2}+(n_{2}-1)s_{D}^{2}}{n_{1}+n_{2}-2}}.$$
  where $s_{C}$ and $s_{D}$ are the standard deviations of Betti numbers for the control and disease groups, and $n_{1}$ and $n_{2}$ are the sample sizes in the control and disease groups, respectively. In our case, $n_{1}=n_{2}=\tau =101,$ corresponding to the number of filtration steps. Cohen’s d standardizes differences across pathways, enabling comparison of the magnitude of effects across different topological features (e.g., disrupted connected components or cycles) and highlighting biologically meaningful changes.

#### 3.6.1. Biological interpretation of the KS test

A small K-statistic value implies that the considered topological structures in the disease and control groups are quite similar, suggesting that the health condition has little influence on the cohesion (or lack thereof) of connected components, or more complex circular patterns, within the pathway network.

A large K-statistic value, instead, indicates that there are noticeable changes between the two groups. For example, for dimension 0 ($\beta_{0}$, connected components), a large KS value could mean that the disease condition is associated with decreased gene connectivity, leading to more disconnected components in the network, potentially reducing the functional output of the pathway. For dimension 1 ($\beta_{1}$, directed cycles), a large KS value could mean that the disease condition potentially affects the cohesion of regulatory loops, like feed-forward loops, destabilizing or enforcing key flow patterns in the pathway.

#### Biological interpretation of cohen’s d

3.6.2

For dimension 0 ($\beta_{0}$, connected components), a positive Cohen’s d means that the control group has a higher number of disconnected components, suggesting genes in the pathway are less co-expressed in the control state compared to the disease group. This could indicate an increased coordination of gene activity in a pathway for the disease condition.

For dimension 1 ($\beta_{1}$, directed cycles), a positive Cohen’s d suggests that network cycles in the control group exhibit tighter co-regulation, whereas, in contrast, in the disease condition the genes involved in these cycles display less coordinated activity.

#### 3.6.3. Criteria for pathway significance

For identifying significant pathway-level differences in Betti numbers between conditions (e.g., control and disease group), we performed KS tests. To evaluate the statistical significance of the obtained KS K-statistic, we conducted 5000 permutations of group labels by randomly assigning the pooled Betti numbers of the disease group series and the control series to one of the two groups and recalculating a KS value for every permutation. We computed the p-value as the proportion of permutations whose calculated K-statistic exceeded the one obtained for the true data. These p-values were corrected for multiple testing across 251 pathways using the BH method, with pathways considered as significant at an FDR <0.05.

Additionally, we applied Cohen’s d to further assess the effect size of differences between conditions for a given pathway. The same permutation process and correction for multiple testing were applied to obtain a list of significantly altered pathways according to Cohen’s d.

Both tests were conducted individually for each dimension of topological features (connected components or cycles). To be more restrictive, we retained only pathways that showed FDR <0.05 for all four tests as significantly altered.

### 3.7. Baseline methods for comparison

As a comparison to our new GenPath-PPH framework, we used three alternative pathway analysis approaches: the PH analysis based on topological descriptors that we presented in a previous study and two topology-unaware methods that are frequently employed by the scientific community.

#### 3.7.1. Persistent homology with topological descriptors (PH-TD)

In our prior work [30], using the same gene expression dataset, we employed PH to identify a set of six topological descriptors (e.g., persistent Euler characteristics, total persistence) that distinguish the PBMC samples of HCC patients from those of healthy controls when constructing point clouds using gene expression profiles from the entire genome. Then, for pathway-specific point clouds (such as those described in Section 3.3), we used the same six descriptors to compare the two health conditions. A pathway was deemed significant if it exhibited statistically significant differences (i.e., FDR <0.05) across all six descriptors, ensuring reproducibility of topological differences.

#### 3.7.2. Hypergeometric Enrichment Analysis (HGEA)

Differential gene expression analysis was performed directly on raw per-gene read counts using the *DESeq2* R package [37] (v1.44.0) to identify genes with significant expression changes between HCC and control groups. Using an FDR threshold of <0.05 and an absolute log_2_ fold change ≥1, we obtained 1251 differentially expressed genes (DEGs): 1095 were up-regulated in the PBMC samples of HCC patients and 156 were down-regulated.

For pathway enrichment, we used a one-sided Fisher’s exact test, as implemented in Python’s *SciPy* (v1.14.1) [36], to identify pathways with a statistically significant over-representation of DEGs. p-values were adjusted for multiple testing across 251 pathways (BH; FDR <0.05). This approach, termed hypergeometric enrichment analysis (HGEA), prioritizes pathways with non-random DEG enrichment.

Algorithm 1 **GenPath-PPH: Integrating Gene Expression and Pathway Networks via Persistent Path Homology**

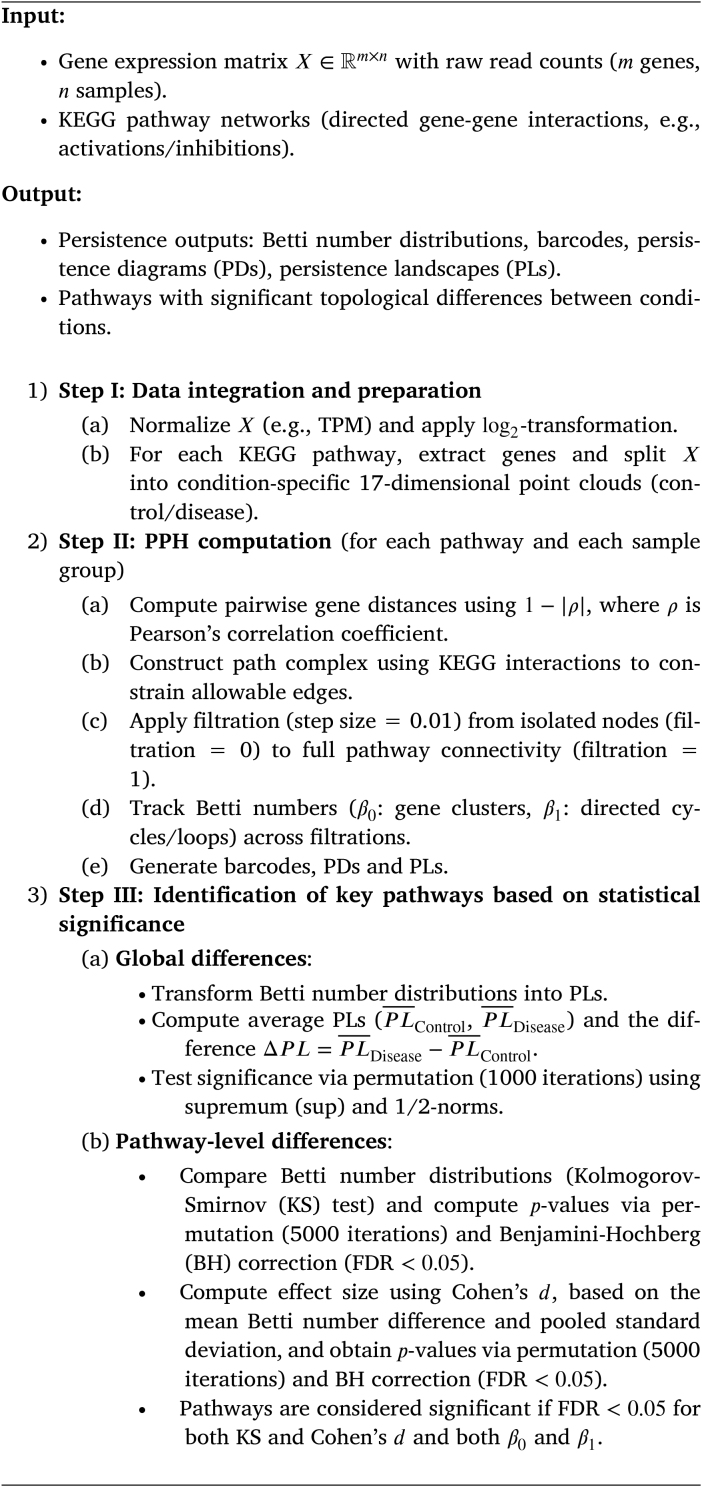

#### 3.7.3. Gene set enrichment analysis (GSEA)

Using the results from *DESeq2* (see above), all genes were ranked by decreasing log_2_ fold change to prioritize those most upregulated in HCC and least expressed in the opposite direction. We then applied GSEA [6] using the *GSEApy* (v1.1.3) Python package [38] to identify pathways enriched with genes at the extremes of this ranked gene list (i.e., highly up- or down-regulated genes). Significance was determined using an FDR threshold of <0.05 from 1000 random permutations of the gene list, ensuring robust detection of pathways with coordinated expression changes.

## 4. Key pathway identification with GenPath-PPH

### 5.1. GenPath-PPH workflow

**Fig. 1.**
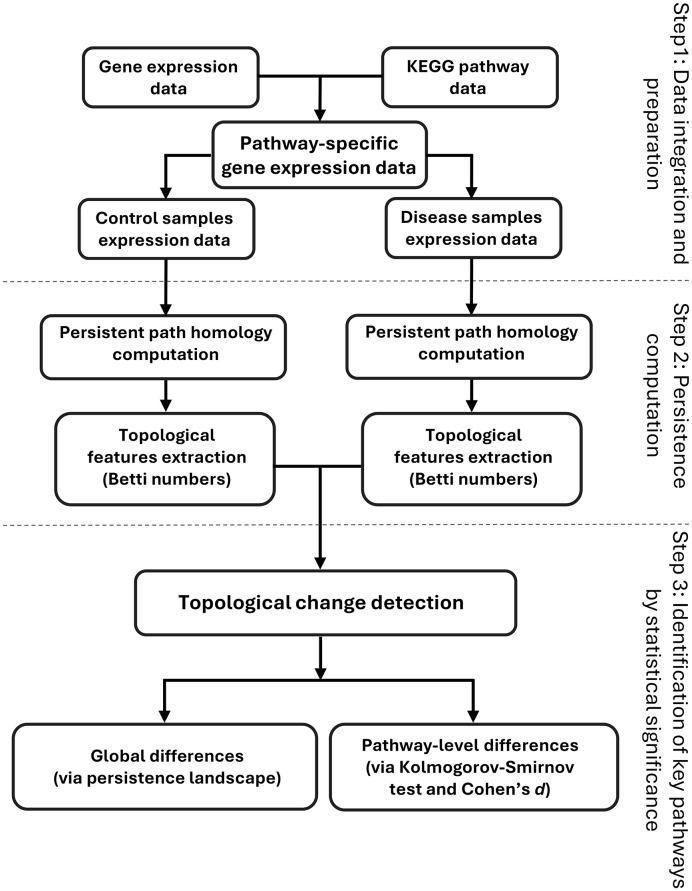
GenPath-PPH workflow.

The GenPath-PPH workflow consists of three stages, which are outlined in the pseudocode in Algorithm 1 and visualized in Fig. 1. A brief explanation of each stage is provided below:

I *Data integration and preparation:* Gene expression data and KEGG pathway information are combined. The gene expression data are normalized (e.g., TPM, used in this study, or other normalization techniques), $\log_{2}$-transformed, and partitioned into pathway-specific, condition-grouped point clouds (control and disease).
II *PPH computation:* Correlation-based gene distances (1−|ρ|, using Pearson correlation) are calculated. KEGG pathway interactions are overlaid to construct directionally constrained path complexes, and edges are incrementally filtered (filtration step size = 0.01) to track Betti numbers ($\beta_{0}$: gene clusters, $\beta_{1}$: directed cycles/loops) across filtrations.
III *Identification of key pathways based on statistical significance:* To detect topological changes, global differences are first quantified via permutation tests on persistence landscapes (PLs) to assess whether the Betti numbers can distinguish between control and disease conditions. Independently, pathway-level topological differences are evaluated using the Kolmogorov-Smirnov test (FDR <0.05) and Cohen’s d (FDR <0.05) for both connected components (dimension 0, $\beta_{0}$) and loops or cycles (dimension 1, $\beta_{1}$). Pathways are considered significant if they meet the FDR threshold (<0.05) in both tests and for both dimensions.

### 4.3. Implementation

The workflow is implemented in a modified version of *PathHom* [22] (available at https://github.com/WeilabMSU/PathHom, accessed on 13 August 2024), with custom functions developed for connectivity tracking, visualization, and statistical analysis. Our code can be found at https://github.com/DrMSAbdullahi/GenPath-PPH. Users can specify pathways in the form of adjacency matrices, which can in principle be taken from databases other than KEGG.

In GenPath-PPH, the filtration step size is fixed at 0.01, providing a balance between detecting relevant topological features and computational efficiency. Larger step sizes may skip features due to having too few filtration levels, whereas smaller step sizes generate many intermediate features, increasing computation time. The filtration scale is also defined based on the distance type (e.g., 1 for (1 - |ρ|), 2 for (1-ρ), or user-defined for Euclidean distance).

Other parameters are flexible and user-configurable, including: the number of permutations, the norm used in PLs, the homology dimensions to track (e.g., $\beta_{0}$, $\beta_{1}$), and the number of samples per condition group. These can be adjusted depending on the dataset and analysis goals.

### 4.3. Illustrative example

**Fig. 2.**
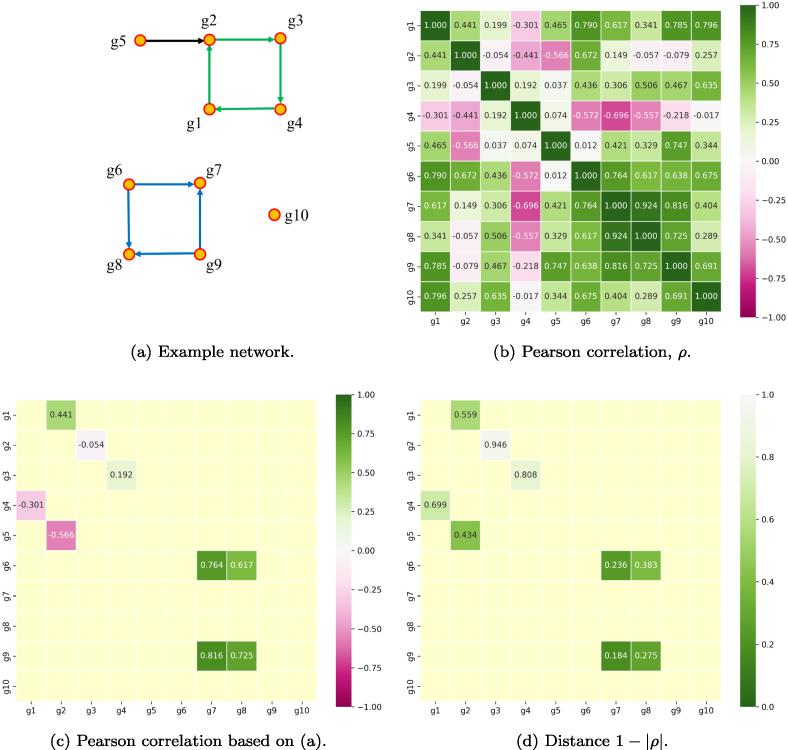
Illustrative toy example for the integration of pathway information and gene expression data for GenPath-PPH. **(a)** Directed gene interaction network (pathway). **(b)** Pearson correlation heatmap for all possible gene pairs, derived from simulated gene expression data. **(c)** Heatmap after gene interactions (edges) present in **(a)** were overlaid, focusing on the correlations of genes that are connected within the network. **(d)** Heatmap showing the distances between connected genes, computed as 1−|ρ|.

To demonstrate how GenPath-PPH addresses limitations of classical PH, we analyze a toy gene network Fig. 2(a) simulating two biologically inspired structures:

1. Cycle 1 (canonical cycle): g1↦g2↦g3↦g4↦g1, represented by the green directed edges.
2. Cycle 2 (directional cross-talk): g6↦g7, g6↦g8, g9↦g7 and g9↦g8, represented by the blue directed edges. This represents directionally constrained interactions (e.g., feed-forward regulatory interactions in signaling pathways).

Apart from these structures, the toy pathway contains two additional genes, a gene connected to cycle 1 (g5) and a completely disconnected one (g10). For all ten genes, we generated simulated gene expression profiles for four samples.

Fig. 2(c) illustrates how for PPH we select only those pairwise co-expression measurements (Pearson correlation coefficients in Fig. 2(b)) that correspond to the directed edges in the pathway network, and Fig. 2(d) shows their conversion to distances 1−|ρ|.

**Fig. 3.**
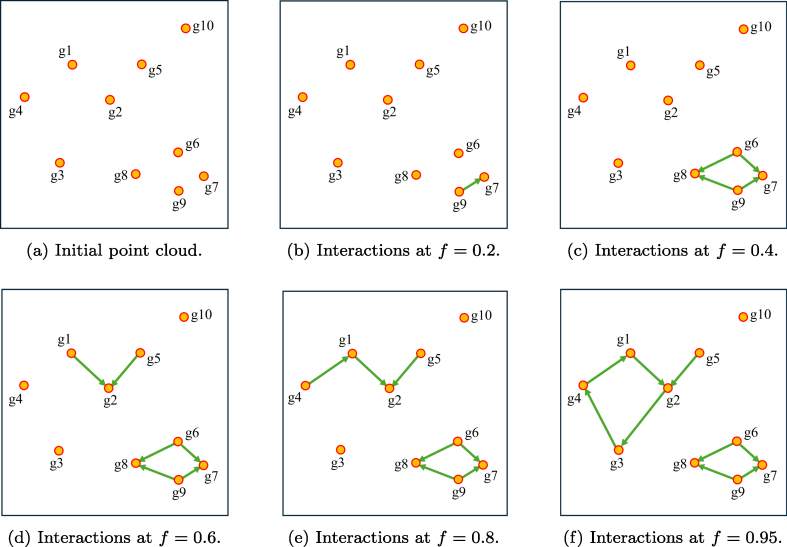
Visualization of the filtration procedure for the toy example from Fig. 2. **(a)** Initial point cloud at a filtration value (distance threshold) of f=0. **(b)**-**(f)** Observed connectivity of genes at various filtration steps, i.e., at various distance thresholds f, obtained using GenPath-PPH. At a threshold of f=0.95 the complete network can be observed because the maximum edge distance is 0.946 (compare to Fig. 2(a) and Fig. 2(d)). GenPath-PPH effectively tracks topological features during the filtration procedure, and at each step topological summaries such as the number of connected components ($\beta_{0}$) and 1-cycles ($\beta_{1}$) are computed.

How the topological features of a directed network can be tracked across increasing filtration values is exemplified in Fig. 3. Filtration values f, which are used as distance thresholds, were increased stepwise from 0 to 1, with a step size of 0.01. At every step, only edges with correlation-derived distances of d=1−|ρ|≤f were considered, starting from the initially disconnected point cloud for f=0 (see Fig. 3(a)). Hence, initially the number of connected components is equivalent to the number of genes in the network ($\beta_{0}$=10) and consequently no 1-cycles can be observed ($\beta_{1}$=0).

Figs. 3(b)–3(f) depict several of the filtration steps and the network edges that are considered at these steps. At filtration f=0.2 (Fig. 3(b)), only the edge g9↦g7 is observed (d=0.184≤f, see Fig. 2(d)), reducing the number of connected components to $\beta_{0}=9$ with still no cycles ($\beta_{1}=0$). At filtration f=0.4 (Fig. 3(c)), one of the two cycles has emerged ($\beta_{1}=1$) because all involved edges have d≤f, and only $\beta_{0}=7$ connected components remain. At f=0.95, finally, all edges of the network are included and the topological summaries stabilize at $\beta_{0}=3$ and $\beta_{1}=2$ Fig. 3(f). Generally, since the maximum possible edge distance d is 1, at the last filtration step f=1, a pathway network will always be observed in its entirety.

An approach based on classical PH, in contrast, would not take the detailed gene interaction information of a pathway into account and would instead use all theoretically possible edges simply according to the distances derived from all the pairwise correlations illustrated in Fig. 2(b). For a description of this approach, we refer to our previous study [30].

**Fig. 4.**
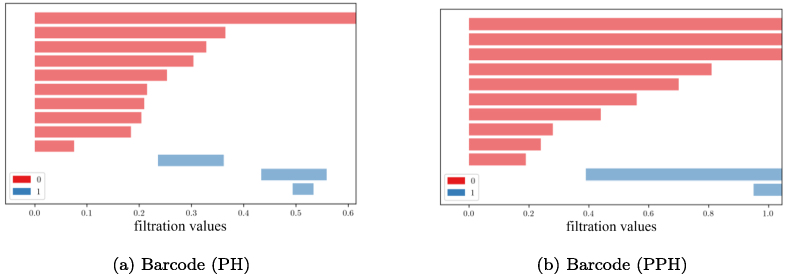
Barcode plots illustrating the persistence of topological features of dimensions 0 (connected components, red) and 1 (cycles, blue), for both **(a)** PH, and **(b)** PPH.

To highlight the conceptual and computational differences between classical PH and direction-aware PPH, we provide a side-by-side illustration in Fig. 4 and explain how each detects topological features.

The barcode plots in Fig. 4 display the persistence of connected components (dimension 0) and cycles (dimension 1) across the filtration procedure for both PH and PPH. For PPH Fig. 4(b), the barplot shows at which filtration thresholds two disconnected components merge because a network edge appears (loss of a red bar, $\beta_{0}$ decreases by one), and at which filtration thresholds cycles become observable because their last edge appears (gain of a blue bar).

In contrast, for PH Fig. 4(a), merging of two components does not depend on the existing network edges; any sufficiently strong correlation (low enough distance) between any two genes is enough for merging. Here, the filtration value f is interpreted as the radius of spheres centered at each data point (gene); components merge when these spheres touch, i.e., when d/2≤f. For these reasons, the number of connected components tends to decrease faster and at $f=d_{\text{max}}/2$ only one large component remains, where $d_{\text{max}}$ is the maximum pairwise distance among genes.

Structural features of dimension 1 also behave differently. In PH, cycles or “holes” appear when the filtration value f is sufficient to let the overlapping filtration spheres of multiple genes or data points form closed bounded structures (e.g., a triangle or a square) but disappear once all spheres overlap completely (see Fig. 4(a)). Because PH does not consider pathway directionality and edge constraints, it can introduce spurious cycles that do not represent true network topology. PPH, by considering connectivity only along directed pathway edges, correctly identifies two cycles in the simulated pathway (see Fig. 2(a) and the blue bars in Fig. 4(b)), whereas PH produces three transient “holes” (see Fig. 4(a)) that do not reflect the true interaction structure.

## 5. Results and discussion

We applied GenPath-PPH to an RNA-seq gene expression dataset of PBMC samples from 17 HCC patients and 17 healthy controls (see Section 3).

### 5.1. Global differences across pathways

**Fig. 5.**
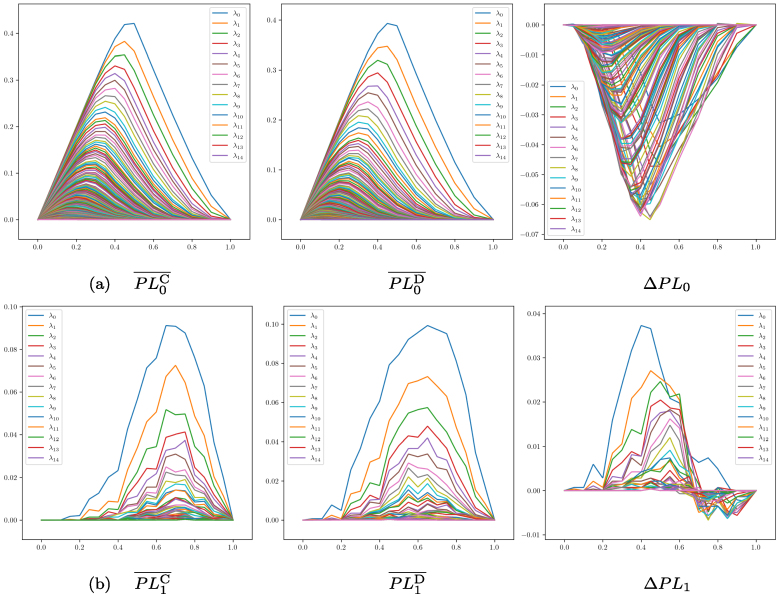
Averaged persistence landscapes (PLs) for **(a)** connected components (dimension 0, top row), and **(b)** 1-cycles (dimension 1, bottom row). For each of the dimensions, the left panel shows averaged PLs for healthy controls, the central panel averaged PLs for HCC-related samples, and the right panel the difference (HCC − control). For each plot only the 15 most dominant landscapes are shown in the legend. C = control group samples, D = disease group samples.

To assess global topological differences between the control and the disease groups, we constructed PLs (see Section 3.5) and computed condition-specific averages over all pathways for dimensions 0 (connected components) and 1 (1-cycles). These averaged PLs are shown in Figs. 5(a) (dimension 0) and 5(b) (dimension 1).

The left panels show PLs for the healthy control samples, where peaks in the landscape functions $\lambda_{k}$ reflect baseline connectivity and cyclic interactions; the central panels display PLs for the samples from HCC patients, revealing altered topology (e.g., fragmentation of connected components or dysregulation of regulatory cycles); and the right panels plot the difference ΔPL, highlighting intervals where HCC-associated $\lambda_{k}$ values exceed or fall below those in controls.

For dimension 0 (connected components; Fig. 5(a)), the negative difference indicates that on average, the control group has more pronounced connectivity (higher landscape peaks) than the disease group, which suggests greater fragmentation of connected components in the latter.

In contrast, for dimension 1 (cycles; Fig. 5(b)), the difference is mixed. At high filtration values (distance thresholds) the difference is slightly negative, but on average and for most of the filtration range, the difference is positive, meaning that the disease group has a stronger co-regulation of cyclic interactions (e.g., regulatory cycles) than the control group.

Permutation tests using different norms (sup-norm for maximum deviation, 1-norm for total absolute difference, and 2-norm for Euclidean distance) confirmed statistically significant differences (FDR <0.05 for all norms), validating PLs as a powerful tool for detecting global topological differences between conditions. The 1-norm captures the overall (global) difference across landscapes, the 2-norm emphasizes larger but distributed deviations, and the sup-norm detects the maximum local deviation at any point. When all norms are significant, as in our case, it implies that differences between control and disease are both globally consistent and locally pronounced. Collectively, these findings demonstrate that PLs are a powerful tool for detecting global topological differences between conditions.

### 5.2. Identification of pathway-level differences

As demonstrated in the previous subsection, the global analysis demonstrates overall class separability, whereas this pathway-level analysis focuses on identifying specific pathways that contribute to (or are altered by) the disease condition.

We used two metrics (KS test statistic and Cohen’s d effect size) to identify significant pathway-level differences. For both metrics, we applied permutation testing to calculate p-values and corrected them for multiple testing (see Section 3.6). Both metrics were independently applied to topological features of dimension 0 (connected components) and those of dimension 1 (cycles).

A pathway was considered significant if its FDR was below 0.05 for both dimensions (0 and 1) and for both metrics. This restrictive approach helped us pinpoint pathways with the most consistent and relevant topological differences in their gene expression networks.

**Fig. 6.**
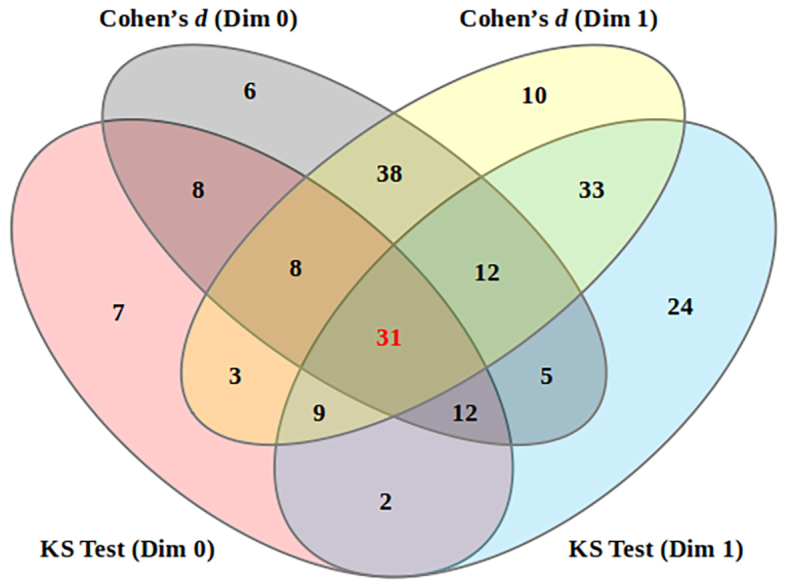
Intersection of pathways identified by the four tests: KS test (Dim 0), KS test (Dim 1), Cohen’s d (Dim 0), and Cohen’s d (Dim 1). Pathways identified by both metrics in both dimensions, considered as significant by GenPath-PPH, are highlighted in red (31 pathways).

In total, 31 pathways were highlighted by GenPath-PPH due to their consistent relevance regarding both metrics and both dimensions (see Table 1). For the overlap of results from the four individual tests, see Fig. 6. Additional details about the pathways identified by each test are provided in the Supplementary Document S1.

**Table 1.** Key metrics for the 31 pathways identified as significant by GenPath-PPH. The table summarizes FDR (KS) and FDR (Cohen’s d) for dimensions 0 and 1. Values marked <0.0002 indicate that the true p-value is smaller than the minimum detectable value based on 5000 permutations. All other values represent the exact FDR obtained.

Pathway	Dimension 0		Dimension 1	
	FDR (KS)	FDR (Cohen’s d)	FDR (KS)	FDR (Cohen’s d)
Aminoacyl-tRNA biosynthesis (hsa00970)	< 0.0002	< 0.0002	< 0.0002	< 0.0002
Ascorbate and aldarate metabolism (hsa00053)	< 0.0002	0.001859	0.049808	0.045216
Complement and coagulation cascades (hsa04610)	0.001931	0.01757	0.001091	0.011202
Fatty acid degradation (hsa00071)	0.035475	0.019959	0.002553	0.000546
Fc epsilon RI signaling pathway (hsa04664)	0.043121	0.031375	0.008367	0.005113
Ferroptosis (hsa04216)	< 0.0002	0.002642	< 0.0002	< 0.0002
Galactose metabolism (hsa00052)	0.019167	0.005193	0.015628	0.003753
Glycosaminoglycan biosynthesis - heparan sulfate /	< 0.0002	< 0.0002	< 0.0002	< 0.0002
heparin (hsa00534)				
Glyoxylate and dicarboxylate metabolism (hsa00630)	0.014953	0.013211	< 0.0002	< 0.0002
Intestinal immune network for IgA production	< 0.0002	0.00873	< 0.0002	< 0.0002
(hsa04672)				
JAK-STAT signaling pathway (hsa04630)	0.031744	0.009323	0.001091	< 0.0002
Lipoic acid metabolism (hsa00785)	0.001931	< 0.0002	< 0.0002	< 0.0002
Lysine degradation (hsa00310)	0.009497	0.025946	< 0.0002	0.030268
Mannose type O-glycan biosynthesis (hsa00515)	0.016733	0.005857	< 0.0002	< 0.0002
NF-κB signaling pathway (hsa04064)	0.014953	< 0.0002	< 0.0002	< 0.0002
Neuroactive ligand-receptor interaction (hsa04080)	0.045116	0.031375	0.004814	0.022609
Nicotinate and nicotinamide metabolism (hsa00760)	0.014953	0.021598	0.018255	0.002953
Pentose phosphate pathway (hsa00030)	0.001931	0.032307	< 0.0002	< 0.0002
Primary bile acid biosynthesis (hsa00120)	< 0.0002	0.037064	< 0.0002	< 0.0002
Prolactin signaling pathway (hsa04917)	0.032012	0.008367	< 0.0002	< 0.0002
Propanoate metabolism (hsa00640)	0.014953	0.043092	< 0.0002	< 0.0002
RNA degradation (hsa03018)	< 0.0002	0.011532	< 0.0002	< 0.0002
Selenocompound metabolism (hsa00450)	0.021137	0.021261	< 0.0002	< 0.0002
Sphingolipid signaling pathway (hsa04071)	0.043121	0.031375	< 0.0002	< 0.0002
Steroid hormone biosynthesis (hsa00140)	0.006972	0.007407	0.003042	0.00108
Synaptic vesicle cycle (hsa04721)	0.028575	0.035992	< 0.0002	0.001569
Terpenoid backbone biosynthesis (hsa00900)	0.006275	0.008367	< 0.0002	< 0.0002
Th17 cell differentiation (hsa04659)	0.006275	0.001859	0.001859	< 0.0002
Thyroid hormone synthesis (hsa04918)	0.016733	0.020917	0.001859	0.025849
Vitamin B6 metabolism (hsa00750)	< 0.0002	< 0.0002	0.010147	0.008152
p53 signaling pathway (hsa04115)	0.023427	0.00873	< 0.0002	< 0.0002

**Fig. 7.**
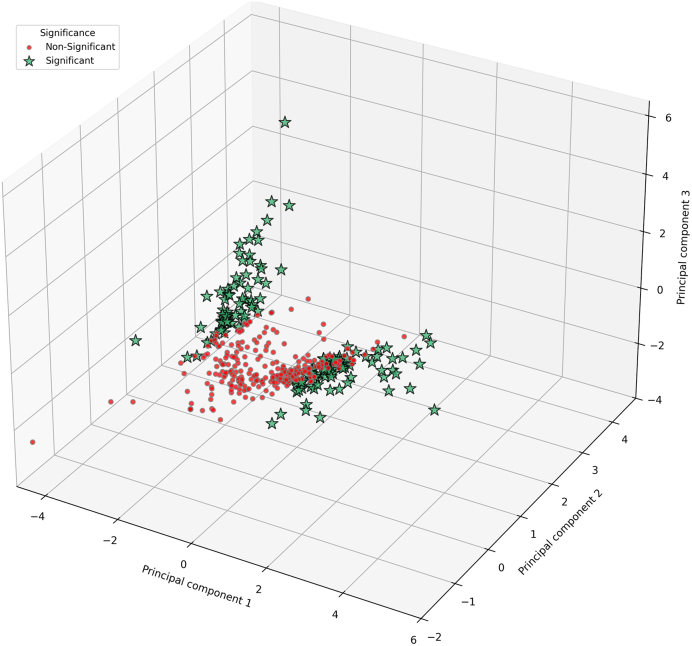
Principal component analysis (PCA) plot illustrating pathway clustering based on GenPath-PPH results. Significant pathways are indicated with green stars and non-significant pathways with red dots.

To validate these findings, we performed principal component analysis (PCA) on multiple pathway-level metrics–KS statistics, mean Betti number differences, and Cohen’s d. PCA reduced these metrics to principal components that capture the main variation across pathways, with the first two principal components explaining 84.1 % of the total variance, while the third component accounted for the remaining 15.9 %. As shown in the 3D plot (Fig. 7), pathways formed two partially distinct clusters that correspond to significant and non-significant groups identified by GenPath-PPH. Clustering robustness was assessed using the silhouette coefficient (0.25), indicating moderate separation. This suggests that the PCA structure reflects meaningful but partially overlapping topological differences between conditions. The multivariate PCA analysis therefore supports the reliability of the pathway-level topological insights provided by GenPath-PPH.

**Fig. 8.**
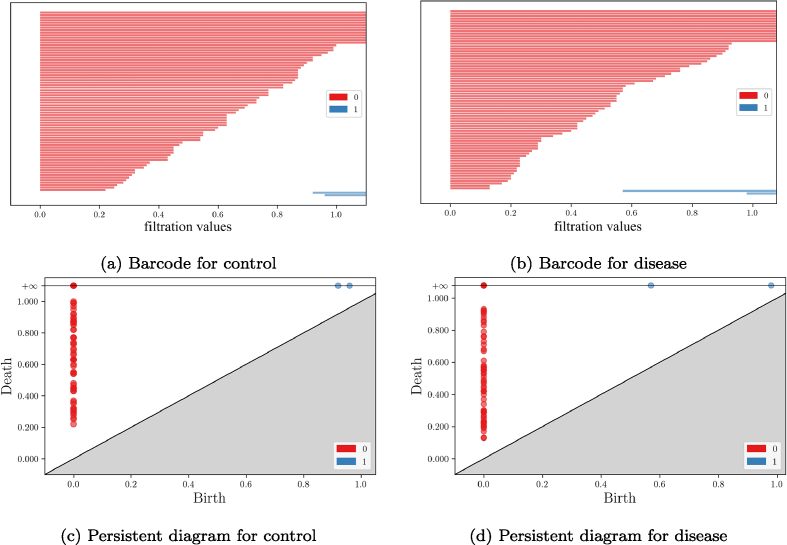
Barcode and PD plots for the p-53 signaling pathway (hsa04115) in control and disease conditions. **(a)** Barcodes for the control group, **(b)** barcodes for the disease group. **(c)** PD for the control group, **(d)** PD for the disease group.

As an illustrative example, the p53 signaling pathway demonstrates how GenPath-PPH captures topological differences between control and disease groups. The barcodes and persistence diagrams in Fig. 8 highlight distinct features in both dimensions. For dimension 0 Fig. 8(a), connected components (red bars) merged earlier in the disease group than in controls. This suggests altered gene connectivity, with a higher fragmentation, i.e., fewer persistent clusters in the disease group. For dimension 1 Fig. 8(b), two 1-cycles (blue bars) emerged at filtration values f=0.58 and f=0.98 in the disease group, compared to f=0.92 and f=0.96 in the control group. The persistence intervals (birth-to-death distances) for these cycles were 0.42 and 0.02 in the disease group, compared to 0.08 and 0.04 in the control group. This suggests a considerably strengthened coordination of one of the cycles in the disease group, i.e., the genes involved in that cycle remain connected over a wider range of similarity thresholds, which indicates a more stable and dominant interaction pattern. These contrasting patterns illustrate how GenPath-PPH detects meaningful differences in both local connectivity and higher-order interactions in pathway structures between conditions.

### 5.3. Comparisons with alternative pathway analysis methods

We compared the pathways identified by GenPath-PPH with those detected by three other methods (PH-TD [30], GSEA, and HGEA). GSEA and HGEA are widely used enrichment-based methods in biological research, while PH-TD is a topological method we previously introduced. PH-TD applies persistent homology to point clouds formed from gene expression similarity but does not account for the structural information of biological pathway networks.

**Fig. 9.**
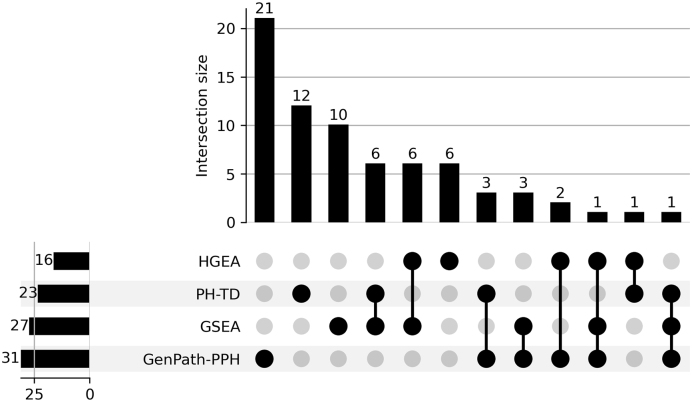
UpSet plot showing the overlap of pathways identified by GenPath-PPH and the three compared existing methods (PH-TD, HGEA, GSEA). The plot highlights unique and shared significant pathways among the methods.

Notably, GenPath-PPH identified 31 pathways with significant topological differences between conditions, compared to 23 detected by PH-TD, 27 by GSEA, and 16 by HGEA (see Table 2). Additionally, GenPath-PPH shared 4 pathways with PH-TD, 5 with GSEA, and 3 with HGEA (see Table 3 for more details). The UpSet plot in Fig. 9 shows limited overlap across the four methods, with no single pathway identified by all of them. This lack of shared pathways mainly results from the three baseline methods (PH-TD, GSEA, and HGEA) not detecting a common set of pathways. These results demonstrate the distinct detection patterns of each method and the complementary insights they provide. To understand these differences, it is important to consider the strengths and limitations of each method.

**Table 2.** Comparison of GenPath-PPH with other methods based on the number and percentage of pathways identified as significant among the total 251 pathways studied.

Method	Pathways identified	
	Number (#)	Percentage (%)
GenPath-PPH	31	12.35
PH-TD (Abdullahi et al. [30])	23	9.16
HGEA	16	6.37
GSEA	27	10.76

**Table 3.** Overlaps of pathways identified by GenPath-PPH with the compared existing methods (PH-TD, HGEA, and GSEA).

Compared Methods	GenPath-PPH
PH-TD	Ascorbate and aldarate metabolism, primary bile acid biosynthesis, synaptic vesicle cycle, p53 signaling pathway
HGEA	Complement and coagulation cascades, JAK-STAT signaling pathway, neuroactive ligand-receptor interaction
GSEA	Complement and coagulation cascades, ferroptosis, galactose metabolism, pentose phosphate pathway, synaptic vesicle cycle

GSEA and HGEA depend on gene-level significance and assume gene independence, which may overlook pathway-level structural organization of genes. PH-TD adopts a topological perspective but is limited to undirected representations of gene expression similarities, and so does not account for the directional nature of biological interactions in the pathway networks. In contrast, GenPath-PPH captures both topological and directional features of gene similarities and pathway structure by integrating the expression data with the directed KEGG pathway graphs using persistent path homology.

One advantage of GenPath-PPH is its ability to detect small changes in topological features across scales that occur within biologically meaningful, directed gene interactions. By restricting analysis to genes with known biological relationships, it avoids noisy signals from unrelated expression patterns. This likely explains why it uniquely identified 21 pathways that were not detected by the other methods. Among these, NF-κB signaling, prolactin signaling, and sphingolipid signaling are well-known to be associated with HCC [39–41]. These pathways contain regulatory motifs such as feedback loops and directionally constrained cascades, which are preserved in KEGG but are often missed by undirected or enrichment-based approaches.

For example, the NF-κB signaling pathway contains several directed cyclic-like structures, including cycles with asymmetric edge orientations, which are detectable using path homology. In both control and disease groups, GenPath-PPH identified three such 1-dimensional cycles. Among them, the first appeared at a lower filtration threshold of 0.5 in the disease group compared to 0.85 in the control group. Since the filtration progresses from 0 (perfect correlation) to 1 (no correlation), this earlier appearance suggests stronger correlations among the genes forming the cycle in the disease condition. Furthermore, all three cycles in the disease group appeared earlier and persisted longer than their counterparts in the control group. This indicates that under disease conditions, coordinated gene interactions not only emerge earlier but also remain stable over a wider range of similarity thresholds, suggesting the presence of stronger and more dominant regulatory motifs. These persistent cyclic structures may reflect sustained or rigid signaling activity. Notably, the NF-κB pathway regulates numerous genes involved in inflammation, cell growth, and apoptosis through direct promoter binding in cancers including HCC [42,43], which may contribute to the formation and stability of these features.

Apart from the well known JAK-STAT, NF-κB, and p53 signaling pathways, many other pathways highlighted by our approach have previously been linked to HCC—either through experimental validation or through indirect evidence, such as the construction of pathway-based risk prognosis models (see Supplementary Document S2). Those pathways that have not yet been directly linked to HCC, or have only indirect support, can therefore be considered as potential candidates.

One of the most promising candidate pathways is aminoacyl-tRNA bio-synthesis. This pathway has been associated with various cancer types, and several aminoacyl-tRNA synthetases are differentially expressed in liver cancer as well [44,45] and show expression levels that correlate with patient survival [45]. Moreover, a recent proteomics study has found the pathway to be enriched in differentially expressed proteins [46]. Although these findings do not directly provide evidence of its involvement in HCC initiation or progression, they are consistent with the importance of the pathway as suggested by our results.

Another interesting candidate is the complement and coagulation cascades pathway. Previous studies have constructed prognostic models based on pathway-related gene signatures that can successfully stratify patients into high- and low-risk groups with distinct survival outcomes [47,48]. Moreover, HCC is found to be associated with a hypercoagulable state driven by liver dysfunction, systemic inflammation, and tumor-related factors [49], which further supports the biological plausibility of this finding.

Altogether, these results suggest that while enrichment-based methods like GSEA and HGEA provide general insight into gene set overrepresentation, and PH-TD offers a complementary topological view based on undirected similarity structures, GenPath-PPH introduces a direction-aware and structure-informed approach. A complete comparison of all the 251 pathways and the detection results from each method is given in Supplementary Document S1 (Table S1).

By integrating pathway structure with gene expression data and focusing only on biologically interacting genes, GenPath-PPH reduces misleading associations from unrelated gene pairs. This approach depends on the quality of the pathway annotations and may miss novel or unrecognized associations, potentially introducing bias toward the pathways represented in the chosen database (e.g., KEGG). The framework can also accommodate other pathway databases, allowing broader application while maintaining sensitivity to biologically relevant pathways shaped by their network structure.

## 6. Conclusion and future research

In this study, we introduced GenPath-PPH, a novel framework that integrates gene expression data with pathway topology using PPH. We applied GenPath-PPH to RNA-seq data of PBMC samples from HCC patients and found it successfully captures structural alterations in biological pathway networks, regarding both connected components and cyclic structures.

These findings are consistent with previous work that demonstrated the utility of persistent homology for synthetic data [50,51]. Our effort successfully extends this to real-world biological networks.

Unlike gene-centric methods (e.g., GSEA, HGEA) or undirected topological analysis (PH-TD), GenPath-PPH uniquely models directed pathway interactions. This enables the detection of important structural changes that other methods might miss. This is also suggested by a comparably low overlap of the identified, significant pathways with those obtained by other methods. Overall, this suggests that GenPath-PPH provides a complementary approach and might be used in concert rather than in opposition to other methods.

Despite its contribution, our study has some challenges. The computational costs, especially with large datasets and complex networks, can be high, which also influenced our choice of a relatively small dataset. Moreover, the sample size in our RNA-seq dataset (17 HCC vs. 17 controls) limits statistical power for detecting subtle pathway differences. To ensure manageable computation and fair comparison between conditions, we used a single dataset with balanced sample sizes as a proof-of-concept. Future work should explore larger and more diverse datasets to evaluate the generalizability and robustness of the framework, potentially applying cross-validation on publicly available HCC cohorts for broader validation.

In addition, extending the framework to other multi-omics data (e.g., proteomics, metabolomics) could broaden its applicability. Future work could also explore the use of alternative pathway databases to increase flexibility across organisms and data sources. Future extensions of GenPath-PPH could also address multi-class settings, such as multiple disease types or varying treatment concentrations, beyond the current pairwise comparison design. Furthermore, integrating the framework (GenPath-PPH) with molecular biology-focused knowledge graphs (KGs) that organize biological entities and relationships across heterogeneous data sources could enhance biological interpretability and hypothesis generation, as demonstrated in recent KG-based studies (e.g., [52]).

## CRediT authorship contribution statement

**Muhammad Sirajo Abdullahi:** Writing – review & editing, Writing – original draft, Visualization, Methodology, Funding acquisition, Formal analysis, Conceptualization. **Rosario Michael Piro:** Writing – review & editing, Supervision, Investigation, Formal analysis. **Apichat Suratanee:** Writing – review & editing, Supervision, Methodology, Investigation, Funding acquisition, Formal analysis. **Kitiporn Plaimas:** Writing – review & editing, Supervision, Methodology, Investigation, Funding acquisition, Formal analysis, Conceptualization.

## Data Availability

The original sequencing data can be accessed at the National Center for Biotechnology Information (NCBI) under project PRJNA739257 at https://dataview.ncbi.nlm.nih.gov/object/PRJNA739257. All original code has been deposited on GitHub at https://github.com/DrMSAbdullahi/GenPath-PPH and is publicly available as of the date of publication.

## Appendix A. Mathematical background

This appendix presents the formal definitions and concepts underlying path homology and persistent path homology. For further details and proofs, we refer the reader to [19,26].

### A.1 Path complexes

Let G=(N,E) be a directed network (digraph) with a vertex set N and directed edge set E⊆N×N. A *path complex* P on N is a collection of ordered vertex sequences (paths)

$$(v_{0},v_{1},\ldots ,v_{n}),$$

such that

(i) every vertex v∈N yields the 0-path (v)∈P;
(ii) whenever $(v_{0},\ldots ,v_{n})\in P$, any truncation obtained by removing the first or last vertex is again in P.

Each subset $P_{n}\subset P$ denotes the collection of all n-paths in P. Hence $P_{0}=N$ and $P_{1}=E.$

We denote by $P_{n}$ the set of allowed n-paths, thus $P_{0}=N$ and $P_{1}=E$. The elements of P are called *allowed elementary paths*.

An *elementary* n*-path* is an ordered sequence $(v_{0},\ldots ,v_{n})\in P_{n}$. Concatenation of paths (when compatible) and truncation preserve membership in the path complex under the admissibility rules above; these algebraic closure properties are the structural foundation for path homology.

Fig. A.10 illustrates the basic structures forming a path complex. Specifically, panel A.10(a) shows the elementary building blocks of path complexes, including 0-paths (individual vertices), 1-paths (edges), and 2-paths (higher-order structures—sequences of consecutive edges). Panel A.10(b) provides examples of triangular configurations. The left structure (T1) forms a simple directed loop or a 1-cycle (from $g_{1}\to g_{2}\to g_{3}\to g_{1}$, with $\beta_{1}=1$), whereas the right structure (T2) forms a directed graph without a conventional closed loop (edges $g_{1}\to g_{2}\to g_{3}$ and $g_{1}\to g_{3}$ with $\beta_{1}=0$) (e.g., feedback-like or sequential interactions among genes). Panel A.10(c) extends this concept to four-node configurations, where some arrangements create closed cycles (with $\beta_{1}=1$) while others remain acyclic (i.e., $\beta_{1}=0$). These examples highlight the variety of connectivity patterns that can exist in directed networks (e.g., cyclic or non-cyclic signaling relationships) and set the stage for defining path homology in subsequent sections.

### A.2 Chain spaces and boundary operator

Fix a field k (typically R or $Z_{2}$ in implementations). For each n≥0, let

$$\Omega_{n}:={\mathrm{span}}_{k}\{(v_{0},\ldots ,v_{n})\in P_{n}\}$$

be the vector space of formal linear combinations of elementary n-paths. The *boundary operator* $\partial_{n}:\Omega_{n}\to \Omega_{n-1}$ is defined by

$$\partial_{n}(v_{0},\ldots ,v_{n})=\sum_{i=0}^{n}{\left(-1\right)}^{i}(v_{0},\ldots ,\hat{v_{i}},\ldots ,v_{n}),$$

extended linearly. The operators satisfy $\partial_{n-1}\circ \partial_{n}=0$, so that the $\left\{\Omega_{n},\partial_{n}\right\}$ form a chain complex

$$\cdots \overset{\partial_{n+1}\;}{\to }\Omega_{n}\overset{\partial_{n}\;}{\to }\Omega_{n-1}\overset{\partial_{n-1}\;}{\to }\cdots \overset{\partial_{1}\;}{\to }\Omega_{0}.$$

### A.3 Path homology groups and betti numbers

The nth *path homology group* is

$$H_{n}:=\ker(\partial_{n})/\mathrm{im}(\partial_{n+1}).$$

Over a field k, the vector space dimension

$$\beta_{n}:=\dim_{k}H_{n}$$

gives the topological invariant called the nth *Betti number*. Specifically, $\beta_{0}$ counts independent connected components (in the path–complex sense) and $\beta_{1}$ counts independent directed cycles (loops). Higher $\beta_{n}$ measure higher-dimensional directed structures when present.

### A.4 Filtrations and persistent path homology (PPH)

A *filtration* is a nested family of subgraphs indexed by thresholds

$$G_{\epsilon_{1}}\subseteq G_{\epsilon_{2}}\subseteq \cdots \subseteq G_{\epsilon_{m}},\;\epsilon_{1}<\epsilon_{2}<\cdots <\epsilon_{m}.$$

Each $G_{\epsilon_{i}}$ induces a path complex $P^{\left(\epsilon_{i}\right)}$ and corresponding chain spaces $\Omega_{n}^{\left(\epsilon_{i}\right)}$ and boundary maps $\partial_{n}^{\left(\epsilon_{i}\right)}$. Inclusion $G_{\epsilon_{i}}↪G_{\epsilon_{j}}$ for $\epsilon_{i}\leq \epsilon_{j}$ induces linear maps on chain complexes and hence linear maps on homology

$$\iota_{i,j}^{n}:H_{n}^{\left(\epsilon_{i}\right)}⟶H_{n}^{\left(\epsilon_{j}\right)}.$$

The sequence of homology vector spaces with the induced maps

$${\left\{H_{n}^{\left(\epsilon_{i}\right)},\;\iota_{i,j}^{n}\right\}}_{i\leq j}$$

is an n-th *persistence module*. For each persistent homology class one records its *birth* and *death* indices: the class appears at $\epsilon_{\text{birth}}$ and becomes trivial or merges at $\epsilon_{\text{death}}$. The associated persistence interval is written

$$[\epsilon_{\text{birth}},\epsilon_{\text{death}}).$$

Over a field, finite persistence modules decompose into interval modules, and this decomposition is represented visually by barcodes or persistence diagrams; the collection of intervals over all n is the persistent path homology of G.

### A.5 Relation to classical (undirected) persistence and computational remarks

Chowdhury and Mémoli show that PPH captures directed asymmetry: in the symmetric (undirected) case the PPH construction reduces to the usual PH of the corresponding symmetric complex. In practice, this means that PPH is sensitive to edge direction and can distinguish directed cycles that classical (undirected) PH may treat as spurious or may not detect correctly.

From a computational perspective, when coefficients are taken over a field, the computation of PPH follows the same algebraic-reduction principles used for standard PH; the worst-case computational complexity remains comparable to that of classical PH algorithms (see [19] for details).

### A.6 Application note

In GenPath-PPH we realize filtrations by thresholding a similarity measure (e.g., absolute Pearson correlation transformed into a distance). Edges that meet the threshold at level $\epsilon_{i}$ are included in $G_{\epsilon_{i}}$; the resulting nested graphs produce induced path complexes whose Betti numbers $\beta_{0}^{\left(\epsilon_{i}\right)},\beta_{1}^{\left(\epsilon_{i}\right)}$ are then tracked across $\epsilon_{i}$ to obtain Betti curves and persistence summaries.

### A.7 Intuitive summary

Classical PH studies how clusters and loops form as connections gradually appear in an undirected network. PPH extends this idea to directed networks by keeping track of the direction of each edge. It detects structures such as feedback loops, signal flows, and other asymmetric relationships that ordinary persistence treats as equivalent. Hence, PPH provides a mathematically consistent way to describe how direction-dependent interactions (for instance, regulatory effects in gene networks) behave across scales.

## Appendix B. Supplementary information

Document S1: Contains lists of pathways identified as significant by each approach (KS test and Cohen’s d for dimensions 0 and 1), along with their overlaps and intersections with existing (baseline) methods used for comparison. It also includes Table S1.
Table S1: A table listing all studied pathways with indications of whether each was identified or not by our approach and the baseline methods.
Document S2: Contains the list of pathways identified as significant by GenPath-PPH, categorized according to the KEGG BRITE pathway classification (e.g., immune-related, metabolic, signaling). Each entry includes the KEGG ID, pathway name, BRITE subclass, indication of its known or potential association with HCC, a brief description of the (possible) relationship, the level of supporting evidence, and relevant PubMed references.
