# Supplementary material for: GenPath-PPH: Integrating gene expression and pathway networks via persistent path homology enhances detection of disease-relevant pathways
Source: Comput Struct Biotechnol J. 2025 Nov 26;27:5348-5362. doi: 10.1016/j.csbj.2025.11.018 (PMC13138103; doi:10.1016/j.csbj.2025.11.018)
Supplement: Multimedia Component 1 [file mmc1.docx]

**Supplementary Material – Document S1**

**Manuscript Title:** GenPath-PPH: Integrating Gene Expression and Pathway Networks via Persistent Path Homology Enhances Detection of Disease-Relevant Pathways

**Authors:** Muhammad Sirajo Abdullahi, Rosario Michael Piro, Apichat Suratanee*, Kitiporn Plaimas*

**Dimension 0 (Cohen’s d, KS Statistics) – (FDR < 0.05) – 59 pathways**

Alanine, aspartate and glutamate metabolism, Aldosterone-regulated sodium reabsorption, Aminoacyl-tRNA biosynthesis, Apoptosis, Ascorbate and aldarate metabolism, Autophagy - other, Biotin metabolism, Cell cycle, Circadian rhythm, Complement and coagulation cascades, Cytosolic DNA-sensing pathway, D-Amino acid metabolism, Drug metabolism - cytochrome P450, Fanconi anemia pathway, Fatty acid degradation, Fc epsilon RI signaling pathway, Ferroptosis, Galactose metabolism, Glycosaminoglycan biosynthesis - chondroitin sulfate / dermatan sulfate, Glycosaminoglycan biosynthesis - heparan sulfate / heparin, Glycosylphosphatidylinositol (GPI)-anchor biosynthesis, Glyoxylate and dicarboxylate metabolism, Hedgehog signaling pathway, Histidine metabolism, Homologous recombination, Intestinal immune network for IgA production, JAK-STAT signaling pathway, Lipoic acid metabolism, Lysine degradation, Mannose type O-glycan biosynthesis, Mucin type O-glycan biosynthesis, NF-kappa B signaling pathway, Neuroactive ligand-receptor interaction, Nicotinate and nicotinamide metabolism, Osteoclast differentiation, Parathyroid hormone synthesis, secretion and action, Pentose phosphate pathway, Peroxisome, Phototransduction, Primary bile acid biosynthesis, Prolactin signaling pathway, Propanoate metabolism, RNA degradation, Selenocompound metabolism, Sphingolipid metabolism, Sphingolipid signaling pathway, Starch and sucrose metabolism, Steroid hormone biosynthesis, Synaptic vesicle cycle, Taste transduction, Terpenoid backbone biosynthesis, Th17 cell differentiation, Thiamine metabolism, Thyroid hormone synthesis, Tyrosine metabolism, Valine, leucine and isoleucine degradation, Vitamin B6 metabolism, mRNA surveillance pathway, p53 signaling pathway.

**Dimension 1 (Cohen’s d, KS Statistics) – (FDR < 0.05) – 85 pathways**

AMPK signaling pathway, Adipocytokine signaling pathway, Amino sugar and nucleotide sugar metabolism, Aminoacyl-tRNA biosynthesis, Ascorbate and aldarate metabolism, Autophagy - animal, C-type lectin receptor signaling pathway, Cellular senescence, Cholinergic synapse, Complement and coagulation cascades, Cortisol synthesis and secretion, Cysteine and methionine metabolism, Cytokine-cytokine receptor interaction, ErbB signaling pathway, Fat digestion and absorption, Fatty acid biosynthesis, Fatty acid degradation, Fatty acid elongation, Fc epsilon RI signaling pathway, Fc gamma R-mediated phagocytosis, Ferroptosis, Folate biosynthesis, FoxO signaling pathway, Fructose and mannose metabolism, GABAergic synapse, Galactose metabolism, Glucagon signaling pathway, Glutathione metabolism, Glycerolipid metabolism, Glycine, serine and threonine metabolism, Glycosaminoglycan biosynthesis - heparan sulfate / heparin, Glycosphingolipid biosynthesis - lacto and neolacto series, Glyoxylate and dicarboxylate metabolism, Growth hormone synthesis, secretion and action, Hippo signaling pathway - multiple species, Inositol phosphate metabolism, Intestinal immune network for IgA production, JAK-STAT signaling pathway, Lipoic acid metabolism, Longevity regulating pathway - multiple species, Lysine degradation, Mannose type O-glycan biosynthesis, Melanogenesis, Mitophagy - animal, NF-kappa B signaling pathway, Necroptosis, Neuroactive ligand-receptor interaction, Nicotinate and nicotinamide metabolism, Notch signaling pathway, Ovarian steroidogenesis, PI3K-Akt signaling pathway, Pantothenate and CoA biosynthesis, Pentose and glucuronate interconversions, Pentose phosphate pathway, Phagosome, Phosphatidylinositol signaling system, Phosphonate and phosphinate metabolism, Primary bile acid biosynthesis, Prolactin signaling pathway, Propanoate metabolism, Protein processing in endoplasmic reticulum, Pyruvate metabolism, RNA degradation, Rap1 signaling pathway, Ras signaling pathway, Selenocompound metabolism, Sphingolipid signaling pathway, Steroid hormone biosynthesis, Sulfur metabolism, Synaptic vesicle cycle, T cell receptor signaling pathway, TNF signaling pathway, Terpenoid backbone biosynthesis, Th1 and Th2 cell differentiation, Th17 cell differentiation, Thyroid hormone synthesis, Tryptophan metabolism, VEGF signaling pathway, Various types of N-glycan biosynthesis, Vascular smooth muscle contraction, Viral protein interaction with cytokine and cytokine receptor, Vitamin B6 metabolism, Wnt signaling pathway, cGMP-PKG signaling pathway, p53 signaling pathway.

**GenPath-PPH (Both Dimensions, Cohen’s d, KS Statistics) - (FDR < 0.05) – 31 pathways**

Aminoacyl-tRNA biosynthesis, Ascorbate and aldarate metabolism, Complement and coagulation cascades, Fatty acid degradation, Fc epsilon RI signaling pathway, Ferroptosis, Galactose metabolism, Glycosaminoglycan biosynthesis - heparan sulfate / heparin, Glyoxylate and dicarboxylate metabolism, Intestinal immune network for IgA production, JAK-STAT signaling pathway, Lipoic acid metabolism, Lysine degradation, Mannose type O-glycan biosynthesis, NF-kappa B signaling pathway, Neuroactive ligand-receptor interaction, Nicotinate and nicotinamide metabolism, Pentose phosphate pathway, Primary bile acid biosynthesis, Prolactin signaling pathway, Propanoate metabolism, RNA degradation, Selenocompound metabolism, Sphingolipid signaling pathway, Steroid hormone biosynthesis, Synaptic vesicle cycle, Terpenoid backbone biosynthesis, Th17 cell differentiation, Thyroid hormone synthesis, Vitamin B6 metabolism, p53 signaling pathway.

**Unique to Dimension 0 (Cohen’s d, KS Statistics) – (FDR < 0.05) – 28 pathways**

Alanine, aspartate and glutamate metabolism, Aldosterone-regulated sodium reabsorption, Apoptosis, Autophagy - other, Biotin metabolism, Cell cycle, Circadian rhythm, Cytosolic DNA-sensing pathway, D-Amino acid metabolism, Drug metabolism - cytochrome P450, Fanconi anemia pathway, Glycosaminoglycan biosynthesis - chondroitin sulfate / dermatan sulfate, Glycosylphosphatidylinositol (GPI)-anchor biosynthesis, Hedgehog signaling pathway, Histidine metabolism, Homologous recombination, Mucin type O-glycan biosynthesis, Osteoclast differentiation, Parathyroid hormone synthesis, secretion and action, Peroxisome, Phototransduction, Sphingolipid metabolism, Starch and sucrose metabolism, Taste transduction, Thiamine metabolism, Tyrosine metabolism, Valine, leucine and isoleucine degradation, mRNA surveillance pathway.

**Unique to Dimension 1 (Cohen’s d, KS Statistics) – (FDR < 0.05) – 54 pathways**

AMPK signaling pathway, Adipocytokine signaling pathway, Amino sugar and nucleotide sugar metabolism, Autophagy - animal, C-type lectin receptor signaling pathway, Cellular senescence, Cholinergic synapse, Cortisol synthesis and secretion, Cysteine and methionine metabolism, Cytokine-cytokine receptor interaction, ErbB signaling pathway, Fat digestion and absorption, Fatty acid biosynthesis, Fatty acid elongation, Fc gamma R-mediated phagocytosis, Folate biosynthesis, FoxO signaling pathway, Fructose and mannose metabolism, GABAergic synapse, Glucagon signaling pathway, Glutathione metabolism, Glycerolipid metabolism, Glycine, serine and threonine metabolism, Glycosphingolipid biosynthesis - lacto and neolacto series, Growth hormone synthesis, secretion and action, Hippo signaling pathway - multiple species, Inositol phosphate metabolism, Longevity regulating pathway - multiple species, Melanogenesis, Mitophagy - animal, Necroptosis, Notch signaling pathway, Ovarian steroidogenesis, PI3K-Akt signaling pathway, Pantothenate and CoA biosynthesis, Pentose and glucuronate interconversions, Phagosome, Phosphatidylinositol signaling system, Phosphonate and phosphinate metabolism, Protein processing in endoplasmic reticulum, Pyruvate metabolism, Rap1 signaling pathway, Ras signaling pathway, Sulfur metabolism, T cell receptor signaling pathway, TNF signaling pathway, Th1 and Th2 cell differentiation, Tryptophan metabolism, VEGF signaling pathway, Various types of N-glycan biosynthesis, Vascular smooth muscle contraction, Viral protein interaction with cytokine and cytokine receptor, Wnt signaling pathway, cGMP-PKG signaling pathway.

**Pathway Analysis from our Previous Paper (PH-TD) – 23 pathways**

ABC transporters, Apelin signaling pathway, Ascorbate and aldarate metabolism, Base excision repair, Citrate/TCA cycle, Collecting duct acid secretion, Drug metabolism - cytochrome P450, Glycine, serine and threonine metabolism, Histidine metabolism, IL-17 signaling pathway, Pantothenate and CoA biosynthesis, Phosphonate and phosphinate metabolism, Porphyrin metabolism, Primary bile acid biosynthesis, Protein processing in endoplasmic reticulum, RNA polymerase, Riboflavin metabolism, Sulfur metabolism, Synaptic vesicle cycle, Tryptophan metabolism, Virion - herpesvirus, beta-Alanine metabolism, p53 signaling pathway.

**Hypergeometric Enrichment Analysis (HGEA) – 16 Pathways**

Calcium signaling pathway, Cholesterol metabolism, Complement and coagulation cascades, Cytokine-cytokine receptor interaction, ECM-receptor interaction, Fat digestion and absorption, Glycerolipid metabolism, Hematopoietic cell lineage, IL-17 signaling pathway, JAK-STAT signaling pathway, Neuroactive ligand-receptor interaction, Neutrophil extracellular trap formation, Osteoclast differentiation, PPAR signaling pathway, Phagosome, Platelet activation.

**Gene Set Enrichment Analysis (GSEA) – 34 pathways**

AMPK signaling pathway, Adipocytokine signaling pathway, Cholesterol metabolism, Collecting duct acid secretion, Complement and coagulation cascades, Drug metabolism - cytochrome P450, ECM-receptor interaction, Ferroptosis, Fructose and mannose metabolism, Galactose metabolism, Glutathione metabolism, Glycine, serine and threonine metabolism, Glycolysis / Gluconeogenesis, Histidine metabolism, Lysosome, Mineral absorption, Natural killer cell mediated cytotoxicity, Neutrophil extracellular trap formation, Olfactory transduction, PPAR signaling pathway, Pantothenate and CoA biosynthesis, Pentose phosphate pathway, Phagosome, Platelet activation, Porphyrin metabolism, Pyrimidine metabolism, Synaptic vesicle cycle.

**GenPath-PPH & PH-TD – 4 pathways**

Ascorbate and aldarate metabolism, Primary bile acid biosynthesis, Synaptic vesicle cycle, p53 signaling pathway.

**GenPath-PPH & HGEA – 3 pathways**

Complement and coagulation cascades, JAK-STAT signaling pathway, Neuroactive ligand-receptor interaction.

**GenPath-PPH & GSEA – 5 pathways**

Complement and coagulation cascades, Ferroptosis, Galactose metabolism, Pentose phosphate pathway, Synaptic vesicle cycle.

**GenPath-PPH (Uniquely identified) – 21 pathways**

Aminoacyl-tRNA biosynthesis, Fatty acid degradation, Fc epsilon RI signaling pathway, Glycosaminoglycan biosynthesis - heparan sulfate / heparin, Glyoxylate and dicarboxylate metabolism, Intestinal immune network for IgA production, Lipoic acid metabolism, Lysine degradation, Mannose type O-glycan biosynthesis, NF-kappa B signaling pathway, Nicotinate and nicotinamide metabolism, Prolactin signaling pathway, Propanoate metabolism, RNA degradation, Selenocompound metabolism, Sphingolipid signaling pathway, Steroid hormone biosynthesis, Terpenoid backbone biosynthesis, Th17 cell differentiation, Thyroid hormone synthesis, Vitamin B6 metabolism.
