# Supplementary material for: GenPath-PPH: Integrating gene expression and pathway networks via persistent path homology enhances detection of disease-relevant pathways
Source: Comput Struct Biotechnol J. 2025 Nov 26;27:5348-5362. doi: 10.1016/j.csbj.2025.11.018 (PMC13138103; doi:10.1016/j.csbj.2025.11.018)
Supplement: Multimedia Component 3 [file mmc3.docx]

**Table S1:** Table showing the identification of pathways by different methods. A cross (×) indicates that a pathway was not identified by the corresponding method, while a red tick (✔) signifies identification. GenPath-PPH identified pathways are highlighted in blue.

| **Pathway** | **GenPath-PPH** | **PH-TD** | **HGEA** | **GSEA** |
| --- | --- | --- | --- | --- |
| Glycolysis / Gluconeogenesis (hsa00010) | 🗶 | 🗶 | 🗶 | ✓ |
| Citrate cycle (TCA cycle) (hsa00020) | 🗶 | ✓ | 🗶 | 🗶 |
| Pentose phosphate pathway (hsa00030) | ✓ | 🗶 | 🗶 | ✓ |
| Pentose and glucuronate interconversions (hsa00040) | 🗶 | 🗶 | 🗶 | 🗶 |
| Fructose and mannose metabolism (hsa00051) | 🗶 | 🗶 | 🗶 | ✓ |
| Galactose metabolism (hsa00052) | ✓ | 🗶 | 🗶 | ✓ |
| Ascorbate and aldarate metabolism (hsa00053) | ✓ | ✓ | 🗶 | 🗶 |
| Starch and sucrose metabolism (hsa00500) | 🗶 | 🗶 | 🗶 | 🗶 |
| Amino sugar and nucleotide sugar metabolism (hsa00520) | 🗶 | 🗶 | 🗶 | 🗶 |
| Pyruvate metabolism (hsa00620) | 🗶 | 🗶 | 🗶 | 🗶 |
| Glyoxylate and dicarboxylate metabolism (hsa00630) | ✓ | 🗶 | 🗶 | 🗶 |
| Propanoate metabolism (hsa00640) | ✓ | 🗶 | 🗶 | 🗶 |
| Butanoate metabolism (hsa00650) | 🗶 | 🗶 | 🗶 | 🗶 |
| Inositol phosphate metabolism (hsa00562) | 🗶 | 🗶 | 🗶 | 🗶 |
| Oxidative phosphorylation (hsa00190) | 🗶 | 🗶 | 🗶 | 🗶 |
| Nitrogen metabolism (hsa00910) | 🗶 | 🗶 | 🗶 | 🗶 |
| Sulfur metabolism (hsa00920) | 🗶 | ✓ | 🗶 | 🗶 |
| Fatty acid biosynthesis (hsa00061) | 🗶 | 🗶 | 🗶 | 🗶 |
| Fatty acid elongation (hsa00062) | 🗶 | 🗶 | 🗶 | 🗶 |
| Fatty acid degradation (hsa00071) | ✓ | 🗶 | 🗶 | 🗶 |
| Steroid biosynthesis (hsa00100) | 🗶 | 🗶 | 🗶 | 🗶 |
| Primary bile acid biosynthesis (hsa00120) | ✓ | ✓ | 🗶 | 🗶 |
| Steroid hormone biosynthesis (hsa00140) | ✓ | 🗶 | 🗶 | 🗶 |
| Glycerolipid metabolism (hsa00561) | 🗶 | 🗶 | ✓ | 🗶 |
| Glycerophospholipid metabolism (hsa00564) | 🗶 | 🗶 | 🗶 | 🗶 |
| Ether lipid metabolism (hsa00565) | 🗶 | 🗶 | 🗶 | 🗶 |
| Sphingolipid metabolism (hsa00600) | 🗶 | 🗶 | 🗶 | 🗶 |
| Arachidonic acid metabolism (hsa00590) | 🗶 | 🗶 | 🗶 | 🗶 |
| Linoleic acid metabolism (hsa00591) | 🗶 | 🗶 | 🗶 | 🗶 |
| alpha-Linolenic acid metabolism (hsa00592) | 🗶 | 🗶 | 🗶 | 🗶 |
| Biosynthesis of unsaturated fatty acids (hsa01040) | 🗶 | 🗶 | 🗶 | 🗶 |
| Purine metabolism (hsa00230) | 🗶 | 🗶 | 🗶 | 🗶 |
| Pyrimidine metabolism (hsa00240) | 🗶 | 🗶 | 🗶 | ✓ |
| Alanine, aspartate and glutamate metabolism (hsa00250) | 🗶 | 🗶 | 🗶 | 🗶 |
| Glycine, serine and threonine metabolism (hsa00260) | 🗶 | ✓ | 🗶 | ✓ |
| Cysteine and methionine metabolism (hsa00270) | 🗶 | 🗶 | 🗶 | 🗶 |
| Valine, leucine and isoleucine degradation (hsa00280) | 🗶 | 🗶 | 🗶 | 🗶 |
| Valine, leucine and isoleucine biosynthesis (hsa00290) | 🗶 | 🗶 | 🗶 | 🗶 |
| Lysine degradation (hsa00310) | ✓ | 🗶 | 🗶 | 🗶 |
| Arginine biosynthesis (hsa00220) | 🗶 | 🗶 | 🗶 | 🗶 |
| Arginine and proline metabolism (hsa00330) | 🗶 | 🗶 | 🗶 | 🗶 |
| Histidine metabolism (hsa00340) | 🗶 | ✓ | 🗶 | ✓ |
| Tyrosine metabolism (hsa00350) | 🗶 | 🗶 | 🗶 | 🗶 |
| Phenylalanine metabolism (hsa00360) | 🗶 | 🗶 | 🗶 | 🗶 |
| Tryptophan metabolism (hsa00380) | 🗶 | ✓ | 🗶 | 🗶 |
| Phenylalanine, tyrosine and tryptophan biosynthesis (hsa00400) | 🗶 | 🗶 | 🗶 | 🗶 |
| beta-Alanine metabolism (hsa00410) | 🗶 | ✓ | 🗶 | 🗶 |
| Taurine and hypotaurine metabolism (hsa00430) | 🗶 | 🗶 | 🗶 | 🗶 |
| Phosphonate and phosphinate metabolism (hsa00440) | 🗶 | ✓ | 🗶 | 🗶 |
| Selenocompound metabolism (hsa00450) | ✓ | 🗶 | 🗶 | 🗶 |
| D-Amino acid metabolism (hsa00470) | 🗶 | 🗶 | 🗶 | 🗶 |
| Glutathione metabolism (hsa00480) | 🗶 | 🗶 | 🗶 | ✓ |
| N-Glycan biosynthesis (hsa00510) | 🗶 | 🗶 | 🗶 | 🗶 |
| Various types of N-glycan biosynthesis (hsa00513) | 🗶 | 🗶 | 🗶 | 🗶 |
| Mucin type O-glycan biosynthesis (hsa00512) | 🗶 | 🗶 | 🗶 | 🗶 |
| Mannose type O-glycan biosynthesis (hsa00515) | ✓ | 🗶 | 🗶 | 🗶 |
| Other types of O-glycan biosynthesis (hsa00514) | 🗶 | 🗶 | 🗶 | 🗶 |
| Glycosaminoglycan biosynthesis - chondroitin sulfate / dermatan sulfate (hsa00532) | 🗶 | 🗶 | 🗶 | 🗶 |
| Glycosaminoglycan biosynthesis - heparan sulfate / heparin (hsa00534) | ✓ | 🗶 | 🗶 | 🗶 |
| Glycosaminoglycan biosynthesis - keratan sulfate (hsa00533) | 🗶 | 🗶 | 🗶 | 🗶 |
| Glycosaminoglycan degradation (hsa00531) | 🗶 | 🗶 | 🗶 | 🗶 |
| Glycosylphosphatidylinositol (GPI)-anchor biosynthesis (hsa00563) | 🗶 | 🗶 | 🗶 | 🗶 |
| Glycosphingolipid biosynthesis - lacto and neolacto series (hsa00601) | 🗶 | 🗶 | 🗶 | 🗶 |
| Glycosphingolipid biosynthesis - globo and isoglobo series (hsa00603) | 🗶 | 🗶 | 🗶 | 🗶 |
| Glycosphingolipid biosynthesis - ganglio series (hsa00604) | 🗶 | 🗶 | 🗶 | 🗶 |
| Other glycan degradation (hsa00511) | 🗶 | 🗶 | 🗶 | 🗶 |
| Thiamine metabolism (hsa00730) | 🗶 | 🗶 | 🗶 | 🗶 |
| Riboflavin metabolism (hsa00740) | 🗶 | ✓ | 🗶 | 🗶 |
| Vitamin B6 metabolism (hsa00750) | ✓ | 🗶 | 🗶 | 🗶 |
| Nicotinate and nicotinamide metabolism (hsa00760) | ✓ | 🗶 | 🗶 | 🗶 |
| Pantothenate and CoA biosynthesis (hsa00770) | 🗶 | ✓ | 🗶 | ✓ |
| Biotin metabolism (hsa00780) | 🗶 | 🗶 | 🗶 | 🗶 |
| Lipoic acid metabolism (hsa00785) | ✓ | 🗶 | 🗶 | 🗶 |
| Folate biosynthesis (hsa00790) | 🗶 | 🗶 | 🗶 | 🗶 |
| One carbon pool by folate (hsa00670) | 🗶 | 🗶 | 🗶 | 🗶 |
| Retinol metabolism (hsa00830) | 🗶 | 🗶 | 🗶 | 🗶 |
| Porphyrin metabolism (hsa00860) | 🗶 | ✓ | 🗶 | ✓ |
| Ubiquinone and other terpenoid-quinone biosynthesis (hsa00130) | 🗶 | 🗶 | 🗶 | 🗶 |
| Terpenoid backbone biosynthesis (hsa00900) | ✓ | 🗶 | 🗶 | 🗶 |
| Caffeine metabolism (hsa00232) | 🗶 | 🗶 | 🗶 | 🗶 |
| Neomycin, kanamycin and gentamicin biosynthesis (hsa00524) | 🗶 | 🗶 | 🗶 | 🗶 |
| Metabolism of xenobiotics by cytochrome P450 (hsa00980) | 🗶 | 🗶 | 🗶 | 🗶 |
| Drug metabolism - cytochrome P450 (hsa00982) | 🗶 | ✓ | 🗶 | ✓ |
| Drug metabolism - other enzymes (hsa00983) | 🗶 | 🗶 | 🗶 | 🗶 |
| RNA polymerase (hsa03020) | 🗶 | ✓ | 🗶 | 🗶 |
| Basal transcription factors (hsa03022) | 🗶 | 🗶 | 🗶 | 🗶 |
| Spliceosome (hsa03040) | 🗶 | 🗶 | 🗶 | 🗶 |
| Ribosome (hsa03010) | 🗶 | 🗶 | 🗶 | 🗶 |
| Aminoacyl-tRNA biosynthesis (hsa00970) | ✓ | 🗶 | 🗶 | 🗶 |
| Nucleocytoplasmic transport (hsa03013) | 🗶 | 🗶 | 🗶 | 🗶 |
| mRNA surveillance pathway (hsa03015) | 🗶 | 🗶 | 🗶 | 🗶 |
| Ribosome biogenesis in eukaryotes (hsa03008) | 🗶 | 🗶 | 🗶 | 🗶 |
| Protein export (hsa03060) | 🗶 | 🗶 | 🗶 | 🗶 |
| Protein processing in endoplasmic reticulum (hsa04141) | 🗶 | ✓ | 🗶 | 🗶 |
| SNARE interactions in vesicular transport (hsa04130) | 🗶 | 🗶 | 🗶 | 🗶 |
| Ubiquitin mediated proteolysis (hsa04120) | 🗶 | 🗶 | 🗶 | 🗶 |
| Sulfur relay system (hsa04122) | 🗶 | 🗶 | 🗶 | 🗶 |
| Proteasome (hsa03050) | 🗶 | 🗶 | 🗶 | 🗶 |
| RNA degradation (hsa03018) | ✓ | 🗶 | 🗶 | 🗶 |
| DNA replication (hsa03030) | 🗶 | 🗶 | 🗶 | 🗶 |
| Base excision repair (hsa03410) | 🗶 | ✓ | 🗶 | 🗶 |
| Nucleotide excision repair (hsa03420) | 🗶 | 🗶 | 🗶 | 🗶 |
| Mismatch repair (hsa03430) | 🗶 | 🗶 | 🗶 | 🗶 |
| Homologous recombination (hsa03440) | 🗶 | 🗶 | 🗶 | 🗶 |
| Non-homologous end-joining (hsa03450) | 🗶 | 🗶 | 🗶 | 🗶 |
| Fanconi anemia pathway (hsa03460) | 🗶 | 🗶 | 🗶 | 🗶 |
| ATP-dependent chromatin remodeling (hsa03082) | 🗶 | 🗶 | 🗶 | 🗶 |
| Polycomb repressive complex (hsa03083) | 🗶 | 🗶 | 🗶 | 🗶 |
| Viral life cycle - HIV-1 (hsa03250) | 🗶 | 🗶 | 🗶 | 🗶 |
| Virion - Human immunodeficiency virus (hsa03260) | 🗶 | 🗶 | 🗶 | 🗶 |
| Virion - Flavivirus (hsa03264) | 🗶 | 🗶 | 🗶 | 🗶 |
| Virion - Lyssavirus (hsa03265) | 🗶 | 🗶 | 🗶 | 🗶 |
| Virion - Herpesvirus (hsa03266) | 🗶 | ✓ | 🗶 | 🗶 |
| Virion - Adenovirus (hsa03267) | 🗶 | 🗶 | 🗶 | 🗶 |
| ABC transporters (hsa02010) | 🗶 | ✓ | 🗶 | 🗶 |
| MAPK signaling pathway (hsa04010) | 🗶 | 🗶 | 🗶 | 🗶 |
| ErbB signaling pathway (hsa04012) | 🗶 | 🗶 | 🗶 | 🗶 |
| Ras signaling pathway (hsa04014) | 🗶 | 🗶 | 🗶 | 🗶 |
| Rap1 signaling pathway (hsa04015) | 🗶 | 🗶 | 🗶 | 🗶 |
| Wnt signaling pathway (hsa04310) | 🗶 | 🗶 | 🗶 | 🗶 |
| Notch signaling pathway (hsa04330) | 🗶 | 🗶 | 🗶 | 🗶 |
| Hedgehog signaling pathway (hsa04340) | 🗶 | 🗶 | 🗶 | 🗶 |
| TGF-beta signaling pathway (hsa04350) | 🗶 | 🗶 | 🗶 | 🗶 |
| Hippo signaling pathway (hsa04390) | 🗶 | 🗶 | 🗶 | 🗶 |
| Hippo signaling pathway - multiple species (hsa04392) | 🗶 | 🗶 | 🗶 | 🗶 |
| VEGF signaling pathway (hsa04370) | 🗶 | 🗶 | 🗶 | 🗶 |
| Apelin signaling pathway (hsa04371) | 🗶 | ✓ | 🗶 | 🗶 |
| JAK-STAT signaling pathway (hsa04630) | ✓ | 🗶 | ✓ | 🗶 |
| NF-kappa B signaling pathway (hsa04064) | ✓ | 🗶 | 🗶 | 🗶 |
| TNF signaling pathway (hsa04668) | 🗶 | 🗶 | 🗶 | 🗶 |
| HIF-1 signaling pathway (hsa04066) | 🗶 | 🗶 | 🗶 | 🗶 |
| FoxO signaling pathway (hsa04068) | 🗶 | 🗶 | 🗶 | 🗶 |
| Calcium signaling pathway (hsa04020) | 🗶 | 🗶 | ✓ | 🗶 |
| Phosphatidylinositol signaling system (hsa04070) | 🗶 | 🗶 | 🗶 | 🗶 |
| Phospholipase D signaling pathway (hsa04072) | 🗶 | 🗶 | 🗶 | 🗶 |
| Sphingolipid signaling pathway (hsa04071) | ✓ | 🗶 | 🗶 | 🗶 |
| cAMP signaling pathway (hsa04024) | 🗶 | 🗶 | 🗶 | 🗶 |
| cGMP-PKG signaling pathway (hsa04022) | 🗶 | 🗶 | 🗶 | 🗶 |
| PI3K-Akt signaling pathway (hsa04151) | 🗶 | 🗶 | 🗶 | 🗶 |
| AMPK signaling pathway (hsa04152) | 🗶 | 🗶 | 🗶 | ✓ |
| mTOR signaling pathway (hsa04150) | 🗶 | 🗶 | 🗶 | 🗶 |
| Neuroactive ligand-receptor interaction (hsa04080) | ✓ | 🗶 | ✓ | 🗶 |
| Cytokine-cytokine receptor interaction (hsa04060) | 🗶 | 🗶 | ✓ | 🗶 |
| Viral protein interaction with cytokine and cytokine receptor (hsa04061) | 🗶 | 🗶 | 🗶 | 🗶 |
| ECM-receptor interaction (hsa04512) | 🗶 | 🗶 | ✓ | ✓ |
| Cell adhesion molecules (hsa04514) | 🗶 | 🗶 | 🗶 | 🗶 |
| Endocytosis (hsa04144) | 🗶 | 🗶 | 🗶 | 🗶 |
| Phagosome (hsa04145) | 🗶 | 🗶 | ✓ | ✓ |
| Lysosome (hsa04142) | 🗶 | 🗶 | 🗶 | ✓ |
| Peroxisome (hsa04146) | 🗶 | 🗶 | 🗶 | 🗶 |
| Autophagy - animal (hsa04140) | 🗶 | 🗶 | 🗶 | 🗶 |
| Autophagy - other (hsa04136) | 🗶 | 🗶 | 🗶 | 🗶 |
| Mitophagy - animal (hsa04137) | 🗶 | 🗶 | 🗶 | 🗶 |
| Cell cycle (hsa04110) | 🗶 | 🗶 | 🗶 | 🗶 |
| Oocyte meiosis (hsa04114) | 🗶 | 🗶 | 🗶 | 🗶 |
| Apoptosis (hsa04210) | 🗶 | 🗶 | 🗶 | 🗶 |
| Apoptosis - multiple species (hsa04215) | 🗶 | 🗶 | 🗶 | 🗶 |
| Ferroptosis (hsa04216) | ✓ | 🗶 | 🗶 | ✓ |
| Necroptosis (hsa04217) | 🗶 | 🗶 | 🗶 | 🗶 |
| p53 signaling pathway (hsa04115) | ✓ | ✓ | 🗶 | 🗶 |
| Cellular senescence (hsa04218) | 🗶 | 🗶 | 🗶 | 🗶 |
| Focal adhesion (hsa04510) | 🗶 | 🗶 | 🗶 | 🗶 |
| Adherens junction (hsa04520) | 🗶 | 🗶 | 🗶 | 🗶 |
| Tight junction (hsa04530) | 🗶 | 🗶 | 🗶 | 🗶 |
| Gap junction (hsa04540) | 🗶 | 🗶 | 🗶 | 🗶 |
| Signaling pathways regulating pluripotency of stem cells (hsa04550) | 🗶 | 🗶 | 🗶 | 🗶 |
| Motor proteins (hsa04814) | 🗶 | 🗶 | 🗶 | 🗶 |
| Regulation of actin cytoskeleton (hsa04810) | 🗶 | 🗶 | 🗶 | 🗶 |
| Hematopoietic cell lineage (hsa04640) | 🗶 | 🗶 | ✓ | 🗶 |
| Complement and coagulation cascades (hsa04610) | ✓ | 🗶 | ✓ | ✓ |
| Platelet activation (hsa04611) | 🗶 | 🗶 | ✓ | ✓ |
| Neutrophil extracellular trap formation (hsa04613) | 🗶 | 🗶 | ✓ | ✓ |
| Toll-like receptor signaling pathway (hsa04620) | 🗶 | 🗶 | 🗶 | 🗶 |
| NOD-like receptor signaling pathway (hsa04621) | 🗶 | 🗶 | 🗶 | 🗶 |
| RIG-I-like receptor signaling pathway (hsa04622) | 🗶 | 🗶 | 🗶 | 🗶 |
| Cytosolic DNA-sensing pathway (hsa04623) | 🗶 | 🗶 | 🗶 | 🗶 |
| C-type lectin receptor signaling pathway (hsa04625) | 🗶 | 🗶 | 🗶 | 🗶 |
| Natural killer cell mediated cytotoxicity (hsa04650) | 🗶 | 🗶 | 🗶 | ✓ |
| Antigen processing and presentation (hsa04612) | 🗶 | 🗶 | 🗶 | 🗶 |
| T cell receptor signaling pathway (hsa04660) | 🗶 | 🗶 | 🗶 | 🗶 |
| Th1 and Th2 cell differentiation (hsa04658) | 🗶 | 🗶 | 🗶 | 🗶 |
| Th17 cell differentiation (hsa04659) | ✓ | 🗶 | 🗶 | 🗶 |
| IL-17 signaling pathway (hsa04657) | 🗶 | ✓ | ✓ | 🗶 |
| B cell receptor signaling pathway (hsa04662) | 🗶 | 🗶 | 🗶 | 🗶 |
| Fc epsilon RI signaling pathway (hsa04664) | ✓ | 🗶 | 🗶 | 🗶 |
| Fc gamma R-mediated phagocytosis (hsa04666) | 🗶 | 🗶 | 🗶 | 🗶 |
| Leukocyte transendothelial migration (hsa04670) | 🗶 | 🗶 | 🗶 | 🗶 |
| Intestinal immune network for IgA production (hsa04672) | ✓ | 🗶 | 🗶 | 🗶 |
| Chemokine signaling pathway (hsa04062) | 🗶 | 🗶 | 🗶 | 🗶 |
| Insulin secretion (hsa04911) | 🗶 | 🗶 | 🗶 | 🗶 |
| Insulin signaling pathway (hsa04910) | 🗶 | 🗶 | 🗶 | 🗶 |
| Glucagon signaling pathway (hsa04922) | 🗶 | 🗶 | 🗶 | 🗶 |
| Regulation of lipolysis in adipocytes (hsa04923) | 🗶 | 🗶 | 🗶 | 🗶 |
| Adipocytokine signaling pathway (hsa04920) | 🗶 | 🗶 | 🗶 | ✓ |
| PPAR signaling pathway (hsa03320) | 🗶 | 🗶 | ✓ | ✓ |
| GnRH secretion (hsa04929) | 🗶 | 🗶 | 🗶 | 🗶 |
| GnRH signaling pathway (hsa04912) | 🗶 | 🗶 | 🗶 | 🗶 |
| Ovarian steroidogenesis (hsa04913) | 🗶 | 🗶 | 🗶 | 🗶 |
| Estrogen signaling pathway (hsa04915) | 🗶 | 🗶 | 🗶 | 🗶 |
| Progesterone-mediated oocyte maturation (hsa04914) | 🗶 | 🗶 | 🗶 | 🗶 |
| Prolactin signaling pathway (hsa04917) | ✓ | 🗶 | 🗶 | 🗶 |
| Oxytocin signaling pathway (hsa04921) | 🗶 | 🗶 | 🗶 | 🗶 |
| Relaxin signaling pathway (hsa04926) | 🗶 | 🗶 | 🗶 | 🗶 |
| Growth hormone synthesis, secretion and action (hsa04935) | 🗶 | 🗶 | 🗶 | 🗶 |
| Thyroid hormone synthesis (hsa04918) | ✓ | 🗶 | 🗶 | 🗶 |
| Thyroid hormone signaling pathway (hsa04919) | 🗶 | 🗶 | 🗶 | 🗶 |
| Parathyroid hormone synthesis, secretion and action (hsa04928) | 🗶 | 🗶 | 🗶 | 🗶 |
| Melanogenesis (hsa04916) | 🗶 | 🗶 | 🗶 | 🗶 |
| Renin secretion (hsa04924) | 🗶 | 🗶 | 🗶 | 🗶 |
| Renin-angiotensin system (hsa04614) | 🗶 | 🗶 | 🗶 | 🗶 |
| Aldosterone synthesis and secretion (hsa04925) | 🗶 | 🗶 | 🗶 | 🗶 |
| Cortisol synthesis and secretion (hsa04927) | 🗶 | 🗶 | 🗶 | 🗶 |
| Cardiac muscle contraction (hsa04260) | 🗶 | 🗶 | 🗶 | 🗶 |
| Adrenergic signaling in cardiomyocytes (hsa04261) | 🗶 | 🗶 | 🗶 | 🗶 |
| Vascular smooth muscle contraction (hsa04270) | 🗶 | 🗶 | 🗶 | 🗶 |
| Salivary secretion (hsa04970) | 🗶 | 🗶 | 🗶 | 🗶 |
| Gastric acid secretion (hsa04971) | 🗶 | 🗶 | 🗶 | 🗶 |
| Pancreatic secretion (hsa04972) | 🗶 | 🗶 | 🗶 | 🗶 |
| Bile secretion (hsa04976) | 🗶 | 🗶 | 🗶 | 🗶 |
| Carbohydrate digestion and absorption (hsa04973) | 🗶 | 🗶 | 🗶 | 🗶 |
| Protein digestion and absorption (hsa04974) | 🗶 | 🗶 | 🗶 | 🗶 |
| Fat digestion and absorption (hsa04975) | 🗶 | 🗶 | ✓ | 🗶 |
| Cholesterol metabolism (hsa04979) | 🗶 | 🗶 | ✓ | ✓ |
| Vitamin digestion and absorption (hsa04977) | 🗶 | 🗶 | 🗶 | 🗶 |
| Mineral absorption (hsa04978) | 🗶 | 🗶 | 🗶 | ✓ |
| Vasopressin-regulated water reabsorption (hsa04962) | 🗶 | 🗶 | 🗶 | 🗶 |
| Aldosterone-regulated sodium reabsorption (hsa04960) | 🗶 | 🗶 | 🗶 | 🗶 |
| Endocrine and other factor-regulated calcium reabsorption (hsa04961) | 🗶 | 🗶 | 🗶 | 🗶 |
| Proximal tubule bicarbonate reclamation (hsa04964) | 🗶 | 🗶 | 🗶 | 🗶 |
| Collecting duct acid secretion (hsa04966) | 🗶 | ✓ | 🗶 | ✓ |
| Glutamatergic synapse (hsa04724) | 🗶 | 🗶 | 🗶 | 🗶 |
| GABAergic synapse (hsa04727) | 🗶 | 🗶 | 🗶 | 🗶 |
| Cholinergic synapse (hsa04725) | 🗶 | 🗶 | 🗶 | 🗶 |
| Dopaminergic synapse (hsa04728) | 🗶 | 🗶 | 🗶 | 🗶 |
| Serotonergic synapse (hsa04726) | 🗶 | 🗶 | 🗶 | 🗶 |
| Long-term potentiation (hsa04720) | 🗶 | 🗶 | 🗶 | 🗶 |
| Long-term depression (hsa04730) | 🗶 | 🗶 | 🗶 | 🗶 |
| Retrograde endocannabinoid signaling (hsa04723) | 🗶 | 🗶 | 🗶 | 🗶 |
| Synaptic vesicle cycle (hsa04721) | ✓ | ✓ | 🗶 | ✓ |
| Neurotrophin signaling pathway (hsa04722) | 🗶 | 🗶 | 🗶 | 🗶 |
| Phototransduction (hsa04744) | 🗶 | 🗶 | 🗶 | 🗶 |
| Olfactory transduction (hsa04740) | 🗶 | 🗶 | 🗶 | ✓ |
| Taste transduction (hsa04742) | 🗶 | 🗶 | 🗶 | 🗶 |
| Inflammatory mediator regulation of TRP channels (hsa04750) | 🗶 | 🗶 | 🗶 | 🗶 |
| Axon guidance (hsa04360) | 🗶 | 🗶 | 🗶 | 🗶 |
| Osteoclast differentiation (hsa04380) | 🗶 | 🗶 | ✓ | 🗶 |
| Longevity regulating pathway (hsa04211) | 🗶 | 🗶 | 🗶 | 🗶 |
| Longevity regulating pathway - multiple species (hsa04213) | 🗶 | 🗶 | 🗶 | 🗶 |
| Circadian rhythm (hsa04710) | 🗶 | 🗶 | 🗶 | 🗶 |
| Circadian entrainment (hsa04713) | 🗶 | 🗶 | 🗶 | 🗶 |
| Thermogenesis (hsa04714) | 🗶 | 🗶 | 🗶 | 🗶 |
| **Total number of significant pathways identified** | **31** | **23** | **16** | **27** |
